# The inbred newt genome unveils molecular mechanisms of behavior, development, and regeneration in urodele amphibians

**DOI:** 10.1016/j.isci.2025.113535

**Published:** 2025-09-09

**Authors:** Yuki Kimura, Miyuki Suzuki, Akinori Okumura, Masatoshi Matsunami, Hiroyo Nishide, Rima Mizuno, Kazuto Bou, Yoshinobu Uno, Tomoaki Nakada, Itaru Hasunuma, Yoshikazu Haramoto, Akimasa Fukui, Takeshi Inoue, Yuki Sato, Katsushi Yamaguchi, Zicong Zhang, Akane Chihara, Mai Takehara, Yuki Shibata, Masaaki Kitada, Nerea Moreno, Ikuo Uchiyama, Yutaka Suzuki, Takashi Takeuchi, Masato Nikaido, Kiyokazu Agata, Atsushi Toyoda, Shuji Shigenobu, Toshinori Hayashi, Ken-ichi T. Suzuki

**Affiliations:** 1 School of Life Science and Technology, Institute of Science Tokyo, Meguro-ku, Tokyo 152-8550, Japan; 2 Division of Biology and Biological Engineering, California Institute of Technology, Pasadena, CA 91125, USA; 3 Trans-Scale Biology Center, National Institute for Basic Biology, Okazaki, Aichi 444-8585, Japan; 4 Graduate School of Medicine, University of the Ryukyus, Ginowan, Okinawa 901-2720, Japan; 5 Department of Basic Biology, The Graduate University for Advanced Studies, SOKENDAI, Okazaki, Aichi 444-8585, Japan; 6 Department of Natural Science, Graduate School of Technology, Industrial and Social Science, Tokushima University, Tokushima 770-8506, Japan; 7 Department of Veterinary Medicine, Nippon Veterinary and Life Science University, Musashino, Tokyo 180-8602, Japan; 8 Department of Biology, Faculty of Science, Toho University, Funabashi, Chiba 274-8510, Japan; 9 Department of Agri-Production Sciences, College of Agriculture, Tamagawa University, Machida, Tokyo 194-8610, Japan; 10 Faculty of Science and Engineering, Chuo University, Bunkyo-ku, Tokyo 112-8551, Japan; 11 Faculty of Medicine, Tottori University, Yonago, Tottori 683-8503, Japan; 12 Department of Anatomy, Faculty of Medicine, Kansai Medical University, Hirakata, Osaka 573-1010, Japan; 13 Institute for the Advanced Study of Human Biology, Kyoto University, Sakyo-ku, Kyoto 606-8501, Japan; 14 Graduate School of Integrated Sciences for Life, Hiroshima University, Higashi-Hiroshima, Hiroshima 739-8526, Japan; 15 Department of Biology, Nippon Medical School, Musashino, Tokyo 180-0023, Japan; 16 Department of Cell Biology, Faculty of Biological Sciences, Complutense University, 28040 Madrid, Spain; 17 Graduate School of Frontier Sciences, The University of Tokyo, Kashiwa, Chiba 277-0882, Japan; 18 Professor Emeritus, National Institute for Basic Biology, Okazaki, Aichi 444-8585, Japan; 19 Comparative Genomics Laboratory, National Institute of Genetics, Mishima, Shizuoka 411-8540, Japan; 20 Hiroshima University Amphibian Research Center, Higashi-Hiroshima, Hiroshima 739-8526, Japan

**Keywords:** Zoology, Phylogenetics, Evolutionary biology, Phylogeny

## Abstract

Salamanders provide excellent models for studying vertebrate evolution, development, and regeneration. To further advance the newt as a model organism in biology, we conducted draft genome sequencing of 20 Gb of an inbred newt (*Pleurodeles waltl*). As part of this study, the *Hoxd11–d13* intergenic region is expanded by over 1 Mb owing to the massive insertion of repetitive sequences including newt-specific satellite DNA. Interestingly, *Myod* and *Bmp4*, genes that are typically involved in vertebrate development, are absent in salamanders. Co-option of *Sodefrin Precursor-like Factor* genes, which encode sex pheromone ligands, suggests a diversification of reproductive behavior among salamanders. Moreover, a limb enhancer of *Shh*, MFCS1/ZRS, retains its function, even though it is positioned approximately 5 Mb away from the promoter. Furthermore, we have identified a functional *cis*-element potentially associated with limb regeneration in this enhancer. The newt genome yields crucial insights into amphibian evolution, behavior, development, and regeneration.

## Highlights

• A newt-specific satellite DNA was identified and characterized
• A gene repertoire unique to salamander behavior, development, and regeneration
• CNEs exhibit robust functionality even with massive repetitive sequence insertion
• A regeneration-associated *cis*-element was found in the *Shh* limb-specific enhancer

## Graphical abstract

**Figure:**
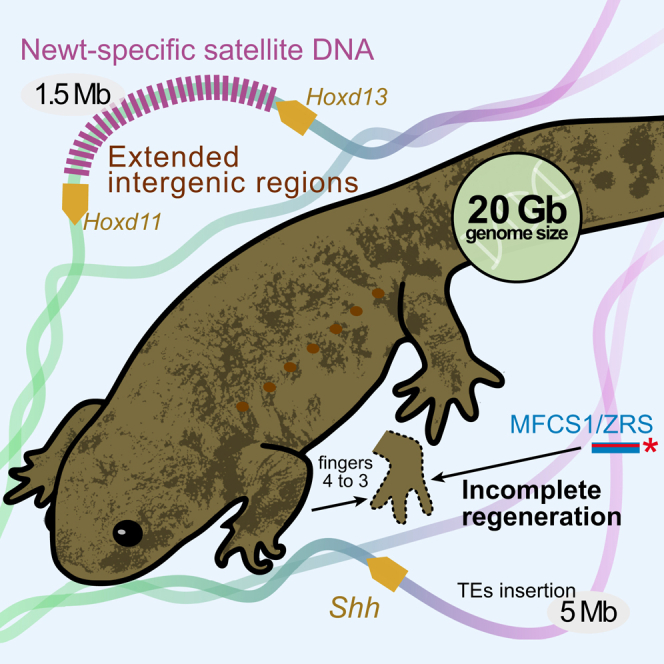

## Introduction

Salamanders, such as newts and the axolotl, have been a subject of significant interest in biology because of their biological characteristics, including organ regeneration discovered by Spallanzani in 1768 and the Spemann-Mangold organizer.^1^ The role of these animals as model organisms offers valuable insights into their remarkable capability for organ regeneration.^2^^,^^3^ However, their exceptionally large genome sizes have presented substantial challenges for genome sequencing, thereby limiting the adoption of advanced molecular biological techniques needed to fully explore their regenerative capabilities.

Recent genome sequencing advances have illuminated the evolutionary signatures underlying unique traits of animals that possess large genomes, including salamanders and lungfishes. The chromosomal-level sequencing of the exceptionally large axolotl genome (*Ambystoma mexicanum*; 32 Gb) was previously reported.^4^ Studies of the genomes of African, Australian, and South American lungfishes (*Protopterus annectens*, *Neoceratodus forsteri*, and *Lepidosiren paradoxa*; 40, 37, and 91 Gb, respectively) have revealed the genomic repertoires associated with water-to-land adaptation, including the fin-to-limb transition.^5^^,^^6^^,^^7^ One of the most notable features of the axolotl and lungfish genomes is their abundance of transposable elements (TEs).^5^^,^^6^^,^^7^^,^^8^ Previous studies have indicated that the large genomes of axolotls and lungfishes have evolved independently from the perspective of their TE repertoires.^5^^,^^6^ Long terminal repeat (LTR) retroelements dominate the axolotl genome, while long interspersed nuclear elements (LINEs) are predominant in the lungfishes’ genomes.^6^^,^^8^ However, the lack of high-quality and long-assembled genomic data from other salamanders has made it difficult to comprehensively compare genomic repertoires among vertebrates, including urodele amphibians. Despite belonging to the same order (Urodela), newts and axolotls exhibit distinct biological characteristics. Newts naturally undergo metamorphosis from larvae to adults, whereas axolotls tend to reach sexual maturity while remaining in a larval form, a phenomenon known as pedomorphosis (neoteny). Consequently, high-quality newt genome data are important for comprehensive analysis of urodele amphibian genomic features.

The Iberian ribbed newt (*Pleurodeles waltl*) is an emerging model organism for studies of physiology, developmental biology, reproductive biology and regenerative biology. This newt possesses a high regenerative capacity in various body parts, including limbs and internal organs, even after metamorphosis. *P. waltl* is an excellent model for reverse genetics using genome editing owing to its ease of maintenance and sexual maturation period of approximately one year.^9^^,^^10^^,^^11^ To establish *P. waltl* as a model organism in biology and enhance our comprehension of salamander evolution and diversity, researchers have long sought a genomic resource for the species. Recently, a chromosome-scale genome project for wild-type *P. waltl* was completed.^12^ In the present study, we performed long-read sequencing and assembly of our established line from repeated laboratory inbreeding (F8 generation) using HiFi read sequencing technology. Here, to complement the previous study,^12^ we present overviews of the genome assembly for the incipient inbred newt line and genomic repertoires unique to amphibians and salamanders in vertebrate evolution, development, and regeneration.

## Results

### Genomic features and statistics of the incipient inbred *P. waltl*

**Figure 1:**
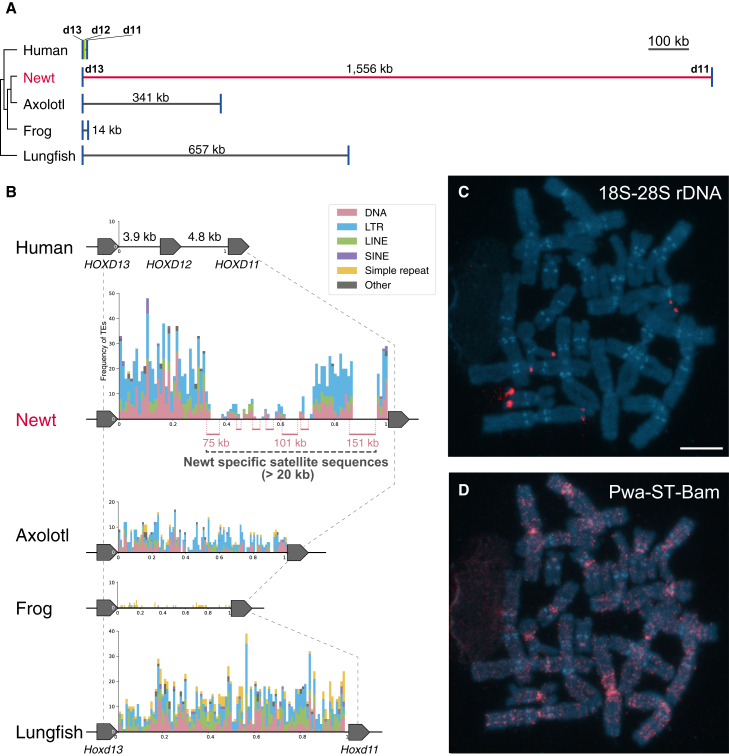
Characterization of a newt-specific satellite DNA in the *P. waltl* genome (A) The lengths of gene intervals between *Hoxd13* and *Hoxd11* have increased independently across different species. (B) Frequencies of TEs in the intergenic region between *Hoxd13* and *Hoxd11*. (C) FISH signals of 18S–28S rDNA are shown for DAPI-stained female metaphase chromosomes. (D) FISH signals of Pwa-ST-Bam for the same metaphase chromosome as in (C). All images were generated by merging multiple photographs owing to the extremely large sizes of newt chromosomes. Scale bar represents 10 μm.

To generate a highly contiguous genome assembly, 650 Gb were sequenced using HiFi-reads technology with approximately 32.5-fold coverage of our own inbred newt genome. Prior to genome assembly, the sequences were profiled using k-mer frequency analysis to assess genomic complexity, including genome size, heterozygosity levels, repeat content, and data quality. Because the newt line used for sequencing was the F8 generation (Female, ZW sex chromosome), it was anticipated that the heterozygosity rate would be relatively low. Heterozygosity rate, genome size, and error rate were estimated using GenomeScope2 (Figure S1).^13^ As expected, the data showed a remarkably low heterozygosity rate of ∼0.07% (Figure S1). The read error rate was 0.09%, indicating high accuracy of our data. The estimated genome size was 20.24 Gb, consistent with previous research.^9^ A total of 2,679 scaffolds were assembled (Table S1). The N50 and N90 were 60.53 and 11.18 Mb, respectively. To evaluate the completeness of our assembly, we used two methods: BUSCO and Merqury.^14^^,^^15^ A BUSCO analysis using the Core Vertebrate Genes (CVG) dataset revealed that the newt genome covered 233 (96.1%) CVG genes (Table S1),^16^ suggesting that this assembly was of considerable quality. The genome completeness in k-mer analysis using Merqury was shown to be 98.58%. The predominance of one-copy k-mers is consistent with the low rate of heterozygosity observed in our GenomeScope2 analysis. These results indicate that the genome sequences for our incipient inbred newt line were sufficiently well reconstructed to retrieve important biological insights. To understand whether the *P. waltl* genome was expanded by TEs as with axolotl and lungfishes, we analyzed repetitive sequences by our analysis pipeline (cTENOR). Repetitive elements comprised 73.3% of the *P. waltl* genome, indicating a high degree of repetitive DNA sequences. The major repetitive elements in the genome were LTR/Gypsy, DNA/Harbinger, and DNA/hAT, which are consistent with previously reported results (described in Figures S2 and S3).^12^ A comparable TE composition was observed in the wild-type *P. waltl* genome, suggesting that the expansion of LTR and DNA transposons is a shared feature across lineages (Figure S2). Similar to previous studies on vertebrate genome gigantism,^5^^,^^6^^,^^8^ we also identified the substantial expansion of Hox clusters in the newt genome (see next section for details). The types and frequencies of repeat sequences in the expanded intergenic region between *Hoxd11* and *Hoxd13* maintained the genome-wide proportion of TEs in which DNA transposons and LTRs are dominant (Figures 1A and 1B). This consistent maintenance of genome-wide repeat composition in the intergenic region between *Hoxd11* and *Hoxd13* was also observed in the axolotl and lungfish genomes (Figures 1B and S2). On the basis of these findings, we suggest that the expansion of the intergenic region in organisms with large genomes occurs because of unbiased TE insertions rather than particular TEs accompanying genome gigantism.

One of our new findings from the repetitive sequence analysis concerns the newt-specific satellite DNAs. We identified a unique satellite DNA that was initially classified as a DNA transposon (classified as “other” in Figure S2); however, subsequent manual BLAST and LAST searches revealed that this sequence is in fact a newt-specific microsatellite (Figure S4A). Our analysis indicates that this microsatellite DNA accounts for 4.3% of the genome. The satellite DNA of *P. waltl* shared 80.9% and 73.9% identity with satellite DNAs of the Japanese fire-bellied newt (*Cynops pyrrhogaster*; Cyp-ST-Bam) and the smooth newt (*Lissotriton vulgaris*; TVm8),^17^ respectively (Figure S4B). A comprehensive analysis of the axolotl and other vertebrate genomes revealed no evidence of a notable BLAST hit for this *P. waltl* satellite DNA. Consequently, we designated it as Pwa-ST-Bam, implying a potential shared origin with Cyp-ST-Bam. Interestingly, a massive insertion of Pwa-ST-Bam was identified in the enlarged intergenic region between *Hoxd11* and *Hoxd13* (Figure 1B). To examine the chromosomal distribution of Pwa-ST-Bam, fluorescence *in situ* hybridization (FISH) mapping was performed for female *P. waltl* chromosomes (ZW). Intense fluorescence signals of Pwa-ST-Bam in the *P. waltl* genome were observed at the centromeric and pericentromeric regions of several chromosomes, while the FISH signals of 18S–28S rDNA as controls were observed to be located in the terminal regions of some chromosomes. Furthermore, weak signals of Pwa-ST-Bam were observed dispersed over the entire genome, present on all chromosomes (Figures 1C and 1D).

Animals with larger genomes have previously been demonstrated to exhibit longer introns compared with non-giant genome species.^5^^,^^6^^,^^8^^,^^12^ In this study, we focused on genes with giant introns. An over-representation analysis (ORA) was conducted on genes with the top 5% largest intron sizes from the genomes of *P. waltl*, axolotl, African lungfish, and human (Figures S5A–S5D). The top two gene ontologies (GOs) commonly enriched in *P. waltl*, axolotl, and lungfish were analogous to the results of ORA in humans (Figure S5E). We identified GOs of genes with long introns uniquely shared by species with giant genomes and revealed that GOs involved in neurotransmission were enriched (Figure S5F). The enlargement of gene bodies is basically caused by the insertion of TEs into introns.^5^^,^^7^ Consequently, gene bodies could be expected to be larger as the number of exons/introns increases. We examined the correlation between gene body size and the number of exons, revealing positive correlations across all species examined (Figure S6).

### Hox cluster architecture in *P. waltl*

**Figure 2:**
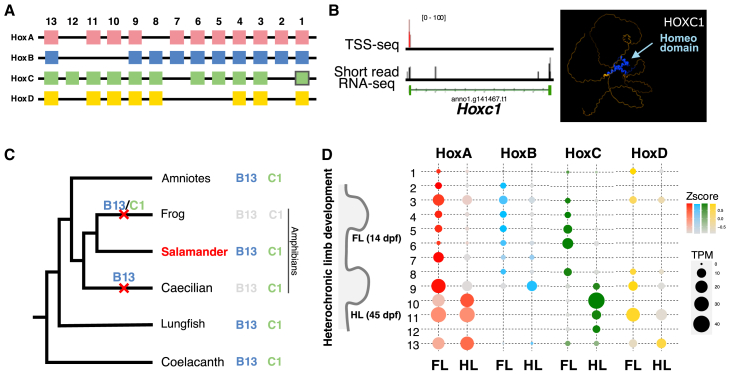
Hox cluster genes and their expression in the *P. waltl* evolutionary development program (A) Organization of Hox cluster genes in *P. waltl*. The four Hox clusters in the *P. waltl* genome possess 40 Hox genes. (B) Characterization of *Hoxc1* gene. Left: mapped read counts of transcriptional start site sequencing (TSS-seq) and RNA-seq at *Hoxc1* gene at the St25 embryo. Right: predicted protein structure of HOXC1 constructed by AlphaFold2. The blue arrow indicates a homeodomain. (C) Presence or absence of *Hoxb13* and *Hoxc1* genes. The cross marks on the branches indicate the presumed timing of the losses of *Hoxb13* and *Hoxc1*. (D) Spatiotemporal collinearity of Hox genes in heterochronic limb development. The circle sizes indicate Transcripts Per Million (TPM) of each *Hox* paralog in forelimbs (FL) and hindlimbs (HL).

The Hox cluster genes play a crucial role in body axis specification and organogenesis in animal evolution.^18^ The HoxA, HoxB, HoxC, and HoxD clusters comprise 40 genes (Figure 2A, Figure S7), and each of the four clusters is completely covered in single contigs in the *P. waltl* genome. In amphibians, Hox genes exhibit distinctive lineage-specific characteristics in *Hoxb13* and *Hoxc1*. *Hoxb13*, absent in *X. tropicalis* (Anura) and *Ichthyophis bannanicus* (Gymnophiona), is retained in both *A. mexicanum* and *P. waltl* (Figures S8 and S9).^19^ The predicted HOXC1 protein has a homeodomain, and its transcript was detected during early embryogenesis (Figure 2B). These findings imply that Hox gene repertoires have been lost asymmetrically across various amphibian orders during their evolution (Figure 2C).^19^

Salamanders, including *P. waltl*, undergo heterochronic limb development: the forelimb develops initially during embryogenesis, followed by the hindlimb, which occurs after hatching. Our analysis revealed that Hox expression of both limb buds shows spatiotemporal collinearity along the body axis (Figure 2D). Anterior Hox genes were predominantly expressed in the forelimb, whereas posterior Hox genes involved in limb development were substantially expressed in the hindlimb. As observed in axolotls and lungfish, the intergenic distances in Hox clusters in the newt are markedly expanded relative to other vertebrates.^5^^,^^8^ In particular, the HoxB/C/D clusters cover an extensive 1–1.5 Mb region (Figure S7). There was no notable expansion of the HoxA cluster in the newt genome exceeding 100 kb, as also seen in the axolotl.^8^ By contrast, other Hox clusters exhibited expansions of intergenic regions surpassing 200 kb, with a tendency to expand more toward the cluster’s ends than its center (Figure S7). The most significant expansion occurred in the HoxD cluster between *Hoxd11* and *Hoxd13*, a distance of 1.5 Mb in a gapless contig (Figures 1A, 1B, and S7). This massive expansion of HoxD is also observed in the axolotl and lungfish.

### Gene repertoire unique to the salamander developmental program and behavior

**Figure 3:**
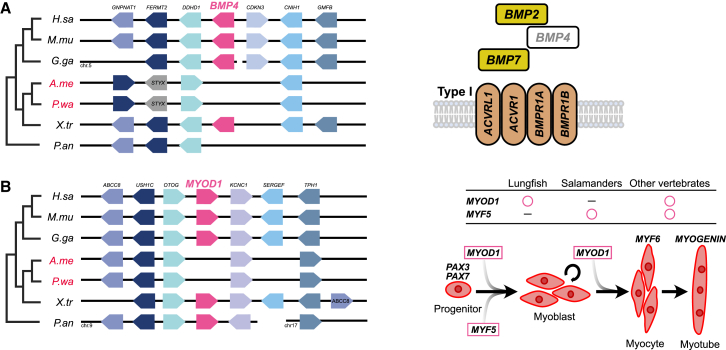
Synteny analysis highlighting gene loss and retention in development-related genes among vertebrates (A) Loss of the *Bmp4* gene (left) and schematic diagram of the TGF-β signaling involved in the genes (right). Note that the *BMP4-like* gene in the African lungfish (*P.an*) is located more than 100 Mb away and is therefore not included in this synteny diagram. (B) Loss of the *Myod* gene (left) and the steps of myogenic differentiation of muscle tissue and the MyoD family genes involved (right).

Most FGF and WNT superfamily genes essential for animal development have been conserved (Figures S10 and S11); however, the newt lacks an otherwise widespread gene from the transforming growth factor β (TGF-β) superfamily. In our previous report concerning gene expression, we proposed that salamanders have lost *Bmp4*, whereas anurans have lost *Bmp16*.^20^ The comparative genome investigation reconfirmed that *Bmp4* is lacking in salamanders at the synteny locus (Figures 3A, S12, and S13). TBLASTX with default parameters searches also identified no orthologs of *Bmp4* in the genome data of *P. waltl* and *A. mexicanum* (Query, *X. tropicalis bmp4*). *Myod* and *Myf5* are bHLH transcription factors that play a central role in muscle differentiation and regeneration/repair processes in animals.^21^ We interrogated the genome assemblies of two salamanders and the African lungfish using TBLASTX and synteny analysis but were unable to detect any orthologs of *Myod* in the salamander genomes or *Myf5* in the lungfish genome (Figures 3B and S13). Conversely, other myogenic factors involved in inducing muscle terminal differentiation, such as *Mrf4/Myf6* and *Myogenin*, were conserved (Figure S14). Pairwise synteny analysis also confirmed that *Bmp4* and *Myod* were absent in both wild-type and inbred *P. waltl*, as well as in axolotl, supporting the interpretation that these may represent genuine gene losses rather than assembly artifacts (Figure S13).

*Nkx*s are homeobox-type transcription factors that are involved in organogenesis, in particular, the pharyngeal arch and its derivatives.^22^^,^^23^ We discovered that *Nkx2.9*, which only occurs in amphibians, was expressed during newt embryogenesis from the neurula to the tail bud stage (Figure S15A). The AlphaFold2 predicted protein structure of NKX2.9 was similar to that of other NKXs (Figure S15B). Additionally, *Nkx3.3* was identified in the genomes of amphibians and lungfish, whereas its presence in amniotes is unlikely (Figure S15C and S15E). *Nkx2.9* and *Nkx3.3* were highly expressed during newt embryogenesis (Figure S15D).

**Figure 4:**
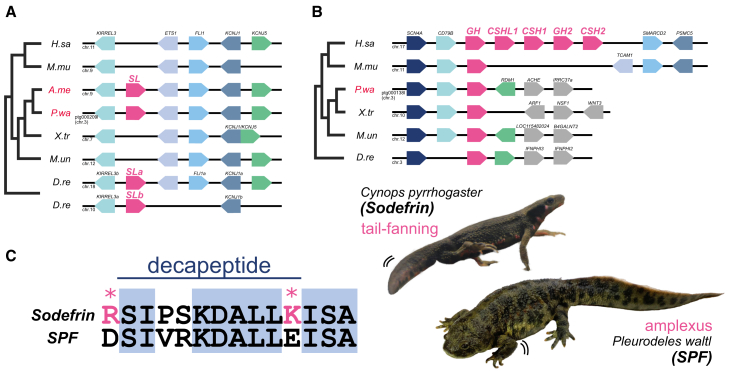
Diversification of reproductive behavior and pheromone-related genes in salamanders (A and B) Somatolactin (*Sl*) genes (A) and growth hormone (*Gh*) genes (B) synteny relationships. Note that *Sl* and *Gh* genes are considered to be derived from a common ancestral gene (Figure S16). (C) C terminus of Sodefrin and Sodefrin Precursor-like Factor (SPF) amino acid sequences between *C. pyrrhogaster* and *P. waltl*. The asterisks indicate the positions of basic amino acid residues (K/R) involved in processing for decapeptide synthesis in *Cynops* Sodefrin (line annotation). In the *P. waltl* SPF, those positions are substituted with acidic amino acid side chains (D/E) and are not subjected to processing. *Cynops* uses Sodefrin and tail-fanning for reproductive behavior, while *P. waltl* performs amplexus and possibly uses the whole SPF protein.

Somatolactin (*Sl*) belongs to the Growth Hormone (*Gh*)/Prolactin (*Prl*) family and has been found only in teleost fish, lungfish, and sturgeons.^24^^,^^25^ The phylogenetic and syntenic analyses revealed the presence of the *Sl* gene in the salamanders (Figures 4A and S16). While the synteny around the *Sl* gene is well conserved among *P. waltl*, *A. mexicanum,* and *Danio rerio* genomes, *Sl* genes have not been identified in anuran (*X. tropicalis*) and caecilian (*Microcaecilia unicolor)* genomes*,* suggesting that only salamanders retain *Sl* genes (Figure 4A). Conversely, *Gh* genes were identified in all amphibians (Figure 4B). *Sl* transcripts were highly expressed from oogenesis through the early stage of embryogenesis in newt (Figure S16).

Sodefrin is a decapeptide pheromone that plays an important role in reproductive behavior and is unique to the genus *Cynops*.^26^^,^^27^ The *Sodefrin* gene product is derived from a precursor protein, Sodefrin precursor-like Factor (SPF)-beta through both frameshift mutation and amino acid substitutions and is released from the abdominal gland in males.^28^^,^^29^ The *Spf* gene family has multiple copies by tandem duplication in *P. waltl*. An interrogation of the whole-genome data revealed that at least 18 *Spf* gene loci existed in four contigs (Figure S17A). C-terminal processing of Sodefrin is essential for producing a decapeptide hormone in *Cynops*, which displays by tail-fanning. By contrast, no processing sites (K/R) were found at the C termini of all SPFs in *P. waltl*, which exhibits embracing as a reproductive behavior (Figure 4C). Their expression was mainly observed in the forelimb blastema and tail including skin, and all of these loci are likely to be functional genes rather than pseudogenes (Figure S17B).

A comparative analysis of the chemoreceptor repertoire was conducted across six families (*OR*s, *TAAR*s, *V1R*s, *V2R*s, *T1R*s, and *T2R*s) among vertebrates, including the wild-type *P. waltl* genome as previously determined (Tables S2–7).^12^ Clear differences in the numbers of chemosensory genes were detected for *V2R*s (incipient inbred, 321; wild-type, 308), particularly in the tetrapod-specific *V2R* (t-*V2R*) subfamily, whereas almost no differences were observed in other chemoreceptor families (Table S6). To determine whether this difference is biological or technical, we performed a phylogenetic analysis of t-*V2R* genes from both newt lines, which revealed a single clade specifically expanded in the inbred line (Figure S18). Notably, the sequences within this clade exhibit minor variations rather than being identical, indicating that the duplicated genes specific to the inbred line are not assembly errors but genuine lineage-specific duplications. Such duplications are commonly observed in olfactory receptors as a mechanism of environmental adaptation, and in this case they appear to have occurred not only between species but also between the wild-type and inbred newt lines.

### Expanded intergenic regions between CNEs and gene bodies in salamander genomes

**Figure 5:**
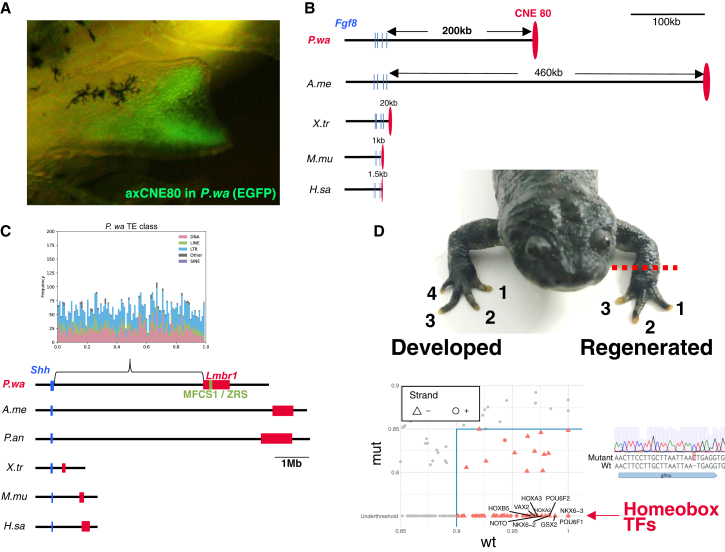
Extended intergenic regions between CNEs and gene bodies involved in the evolutionary development and regeneration programs (A) CNE80 enhancer activity in the transgenic newt. EGFP reporter expression is observed in the forelimb mesenchyme during embryogenesis. (B) Comparison of the distance between CNE80 and the *Fgf8* gene across vertebrates. (C) Comparison of the distance of the *Shh* gene to MFCS1/ZRS. Top: The histogram shows frequency of transposable elements in the genomic region between MFCS1/ZRS and the *Shh* gene body. Bottom: MFCS1/ZRS is located within intron 5 of the *Lmbr1* gene across vertebrates. (D) The phenotype of an MFCS1/ZRS mutant newt (F2). Top: The right forelimb was unamputated as a control (four digits), and the left forelimb was regenerated with a defect (three digits) 1 year post amputation. Bottom left: Candidates of transcription factors bound to a novel regeneration-associated *cis*-element analyzed by JASPAR. Bottom right: Chromatogram and sequence of the mutant (above) and wild-type sequence (below). A one-base insertion is labeled in red.

As Conserved Non-coding Elements (CNEs) are evolutionarily conserved transcriptional control sequences, most of their changes and functions are also constrained in basic vertebrate development. The CNE functions related to limb, heart, and lung development are conserved even with genome enlargement. For example, axCNE80, a limb enhancer of the *Fgf8* gene found in the axolotl,^4^ is well conserved and exhibits the same limb-specific transcriptional activity in a transgenic newt (Figures 5A and S19A). The distance between CNE80 and the *Fgf8* gene body was found to be expanded to a range of 200–460 kb in salamanders, with the expansions representing a magnitude of 20–40 times greater than that observed in other vertebrates (Figure 5B). *Tbx5* is involved in heart development, and its knock-out causes abnormalities in heart development across vertebrates, including newts.^10^ A highly conserved CNE of *Tbx5*, Enh9, which is relevant to human congenital heart disease,^30^ was located 400 kb away from the transcription start site in the newt genome (Figure S20A). Conversely, certain CNEs located within introns appear to remain relatively close to their associated promoters. The *Tbx4* gene plays a role in lung development, and its lung-mesenchyme enhancer is highly conserved across vertebrates (LME, Figure S20B).^31^ This CNE is located in the intron of each gene body; however, it was not located at the far distances observed with the limb-specific CNEs of genes such as *Shh*.

*Shh* plays a crucial role in limb development, and its limb-specific enhancer, MFCS1/ZRS, is located at the intron 5 of *Lmbr1* across vertebrates. This CNE is well known as an ultra-distal enhancer that functions approximately 1 Mb apart from the *Shh* promoter in humans and mice.^32^ The newt, lungfish, and axolotl genome data revealed that the expansion of the intergenic region between *Shh* and *Lmbr1* exhibits an extremely long expansion, reaching up to 5 Mb (Figures 5C and S19B). In our previous study, we obtained various newt mutant lines in which MFCS1/ZRS is disrupted by CRISPR-Cas9. As in other vertebrates, this enhancer is also essential for *Shh* function in newt limb development,^10^ even though it is located as far as 5 Mb away from the gene body (Figure S21). Intriguingly, we found a single *cis*-element related to limb regeneration from one of these mutant lines. Newts with a 1 bp insertion near the snake deletion site in the MFCS1/ZRS enhancer,^33^ which is homozygous, exhibited normal limb development. However, all regenerated limbs displayed digital defects (digit loss from four to three digits in the forelimb; Figures 5D, S21 and S22). We predicted candidates of transcription factors bound to the *cis*-element around the location where this insertion occurred. All of the most likely candidates were homeobox-type transcription factor genes (Figure 5D; TAATTA).

## Discussion

### Transposon proportions and newt-specific satellite DNAs as drivers of genome gigantism

In addition to the chromosome-level genome data of a wild-type *P. waltl* reported by Brown et al.,^12^ in this study we determined the genome of an incipient inbred *P. waltl* line with remarkably low heterozygosity (Table S1, Figure S1). In salamanders, TEs have been shown to be a main component of their giant genomes.^8^^,^^9^^,^^34^ The comparison between *P. waltl* and axolotl revealed that the newts have a smaller genome size but an increased proportion of DNA transposons (Figure S2). Given that the predominance of each DNA transposon family is similar in both species, our findings indicate that general DNA transposon activity has been higher in the newt lineage after divergence from the common ancestor of salamanders (Figure S3A). In a previous study, the rate of DNA loss was estimated to be lower in salamanders than in other vertebrates.^35^ This suggests that the difference in genome size between newt and axolotl may be due to variation in the rate of DNA loss or TE activity.

The composition of TEs in lungfish with large genomes was shown to be substantially different from that of salamanders, which is consistent with previous studies (Figure S2).^5^^,^^6^^,^^7^ The genomes of salamanders also differ substantially from those of other amphibians in both genome size and TE composition, suggesting that genome size enlargement and TE expansion occurred independently in the salamander lineage (Urodela). As a significant portion of the newt genome consists of DNA transposons and LTRs (Figure S2), and DNA transposons are believed to amplify via translocation during DNA replication,^36^ an increase in the length of the DNA strand and replication time would result in an elevated probability of translocation. Future analyses of the TE family expansion dynamics across urodeles, as more genomes become available, will help clarify the evolutionary basis of genome size variation.

A scattering of newt-specific microsatellites (Pwa-ST-Bam) was observed (Figure 1D), comprising 4.3% of the newt genome. These microsatellites may be responsible for the variation in the length of intergenic regions within newts, as seen in the HoxD cluster (Figure 1B). Cyp-ST-Bam, which is the satellite DNA of *C. pyrrhogaster,* is located on the centromeres in all chromosomes of *C. pyrrhogaster*.^17^ However, Pwa-ST-Bam was found not only in centromeres but also dispersed throughout the entire genome (Figure 1D). These results suggest that Pwa-ST-Bam had amplified over the entire genome, including the HoxD cluster, independently in the lineage of *P. waltl* after divergence from the common ancestor of Salamandridae 56 million years ago.^37^ These findings raise intriguing questions about the origin, expansion, and potential function of Pwa-ST-Bam. Future studies comparing chromosomal structures across newt species and salamanders, as well as chromatin conformation analyses such as Hi-C, may help clarify how this satellite DNA spread throughout the *P. waltl* genome after divergence from the common ancestor of Salamandridae, and whether it contributes to large-scale genomic architecture or chromatin organization.

Genome size and intron length have been shown to be positively correlated.^5^^,^^6^^,^^7^^,^^12^ In the newt, we found a positive correlation between the number of the exons and length of each gene (Figure S6A). The result is consistent with the expectation that TE insertion into introns contributes to the expansion of gene length.

### Gene repertoire unique to the salamander developmental program

To complement a previous study,^12^ we performed a detailed analysis of the genome data obtained from our newly established incipient inbred line. This analysis provided novel insights into the distinctive gene sets involved in urodele amphibian development. Owing to evolutionary constraints, most development-related genes essential for the vertebrate body plan have been conserved. However, in this study, we revealed that certain genes were lost, duplicated, or gained in salamanders (Figure 3). Among these is the TGF-β superfamily, one of the most significant groups of morphogenetic genes. Although *Bmp4* is required for mesoderm induction in mice,^38^ the lack of *Bmp4* has no effect on development or regeneration in salamanders, suggesting that an alternative TGF-β signaling pathway may be at work (Figure 3A).^38^^,^^39^ Both *Myod* and *Myf5*, master regulator myogenic transcription factors, drive the differentiation of mesenchymal stem cells into muscle progenitor cells.^40^^,^^41^ Intriguingly, the absence of *Myod* in salamanders and the possible absence of *Myf5* in African lungfish may enhance regenerative capacity, by facilitating dedifferentiation and maintenance of an undifferentiated state during organ regeneration (Figure 3B). In vertebrates, *Myod* induces fibroblasts to become myofibroblasts, promoting overproduction of extracellular matrices and fibrosis during the process of wound healing.^42^ Thus, the absence of *Myod* could be crucial for suppressing fibrosis and facilitating organ regeneration in highly regenerative salamanders, yet it remains a hypothesis lacking direct evidence. Detailed RNA sequencing (RNA-seq) analyses aligning one-to-one orthologs between *P. waltl* and *Xenopus* during limb regeneration and embryogenesis could elucidate compensatory expression changes in pathways affected by loss of *Bmp4* and *Myod* (e.g., TGF-β signaling and myogenesis) and should provide insights into the divergent developmental process and regenerative capacities of urodeles versus anurans, which offers an important direction for future research. Functional validation via gene knockout in *Xenopus*, which retains *Bmp4* and *Myod*, will be critical to determine whether the absence of these genes enhances the regenerative capacity observed in salamanders.

Ultra-long contigs based on highly accurate HiFi sequence data identified all Hox cluster genes in the newt genome (Figures 2 and S7). Among these, the identification of the *Hoxc1* and *Hoxb13* genes revealed asymmetric loss of Hox genes associated with amphibian evolution, suggesting that the common ancestor of amphibians possessed both *Hoxc1* and *Hoxb13* genes (Figure 2). Hox genes located at the terminal ends of each cluster may more easily evade cluster regulation, potentially making them more susceptible to gene loss. Importantly, *Hoxd13*, located at the distal ends of each Hox cluster, plays a critical role in development of appendages such as tails and limbs.^43^^,^^44^^,^^45^ Similarly, in the axolotl and lungfishes, a massive expansion of intergenic regions between *Hoxb/c/d13* and other paralogs in each cluster was observed (Figure S7). Because of intergenic expansion in the salamander and lungfish genomes, long-range enhancer sharing and cluster transcriptional regulation are disrupted, causing changes in the expression domains of *Hoxd13* genes in the limbs.^5^^,^^46^

*Nkx* transcription factors are crucial for the development of the heart, nervous system, and pharyngeal arch derivatives such as the jaw and gill in amphibians and fishes. In deuterostome genomes including those of axolotl and newt, pharyngeal arch clusters consist of the ordered *Nkx2, Pax1/9,* and *Foxa* genes.^22^^,^^47^ *Nkx2.9* and *Nkx3.3* have been identified in the genomes of amphibians, with *Nkx3.3* also present in lungfish (Figure S15). Both genes are located outside of the two pharyngeal arch gene clusters in the newt genome. These orthologs are expressed in the branchial arches of *Xenopus* during embryogenesis, suggesting that these *Nkx* genes may contribute to the formation of internal and external gills in amphibians and fish.^48^^,^^49^ Future functional studies, such as loss-of-function analyses, will be important to clarify the roles of these retained genes in amphibian development and regeneration.

### Reproductive behavior gene repertoires in the salamanders

The present study also provided novel findings involved in salamander reproductive behavior. Growth hormone (*Gh*) and somatolactin (*Sl*) genes are considered to be derived from a common ancestral gene; however, *Sl* was secondarily lost in terrestrial tetrapods.^50^ In teleost fishes, *Sl* is thought to regulate body color, lipid metabolism, and cortisol secretion.^51^^,^^52^^,^^53^^,^^54^ Body color is a visual cue that affects the success rate of reproduction in some salamanders.^55^ Conservation of the *Sl* gene in *P. waltl* possibly relates to body color and reproductive behavior (Figure 4A). To understand the contribution of gene repertoires for the diversification of reproductive behavior, further investigation into the relationship between SL signaling and reproductive behavior via body color in salamanders is needed.

In salamander genera other than *Cynops*, whole SPF proteins function as sex pheromones, whereas in *Cynops* the decapeptide sodefrin acts as a pheromone.^26^^,^^56^ Supporting this hypothesis, all the predicted SPF amino acid sequences obtained from the *P. waltl* genome data do not contain any sodefrin-like decapeptide sequences (e.g., K/RSIPSKDALLK/R), which should be conserved in *Cynops* including the lysine (K)/arginine (R) at the processing site (Figure 4C). Therefore, whole SPF proteins would function as courtship pheromones in *P. waltl* and *A. mexicanum*,^57^ rather than a processed short peptide. These results suggest a unique evolution of the *Spf* gene family in the salamanders. Because *Cynops* males attract females by tail-fanning to spread sex pheromones far away, it is advantageous for sodefrin to be a relatively low-molecular-weight compound such as a decapeptide. By contrast, *P. waltl* males embrace the female from behind (amplexus) in reproductive behavior (Figure 4C).^56^ Therefore, the whole SPF protein could possibly be used as a sex pheromone in those salamanders that perform amplexus, rather than the highly diffusible peptide. Co-option of the sex pheromone gene family is considered to be an evolutionary adaptation to diverse reproductive behaviors in the salamanders.^58^ Indeed, expansion of the number of *Spf* genes was observed in the newt genome (Figure S17). We therefore hypothesize that one of these genes was co-opted into a sodefrin-like decapeptide pheromone during salamander evolution, which has been implicated in the regulation of *Cynops*-specific reproductive behavior. Additionally, the copy number variation of *V2R* genes was observed (Table S6). Given that some *V2R*s were shown to receive peptide pheromones in mice,^59^ it is plausible that the difference in the repertoire of *V2R* genes between these two newts might reflect SPF-mediated incipient speciation. Further analysis of receptor ligands and their behavioral responses will help clarify the potential role of these genetic variations in newt speciation.

### Balancing evolutionary constraints and genome expansion in salamanders

Genome expansion has been associated with an increased distance between CNEs and gene bodies in organisms such as salamanders and lungfishes, adding complexity to the regulation of gene expression during development. Our study revealed that, in newts, the MFCS1/ZRS enhancer—which controls *Shh* expression for limb development—is situated extraordinarily far from the *Shh* gene, approximately 5 Mb away, which is five times the distance observed in other vertebrates (Figures 5C, S21, and S22). Despite this substantial separation, this CNE accurately regulates *Shh* expression, executing the limb development program in newts. With genome expansion, the widening gap between CNEs and target genes could potentially destabilize or alter gene expression. For instance, within the HoxD gene cluster, genome expansion disrupts enhancer sharing, possibly altering gene expression patterns of *Hoxd13* in salamanders and lungfishes.^6^^,^^9^^,^^46^ While such changes may contribute to the evolution of novel functions, they also risk altering established gene networks essential for the basic body plan in vertebrates. Nevertheless, the conservation of developmental programs suggests that critical gene regulation by CNEs has been preserved under strong evolutionary constraints, maintaining essential functions across vertebrate evolution.

To counteract the potential destabilizing effects of genome expansion, two mechanisms are likely at work in salamanders. First, topologically associating domains (TADs) play a pivotal role. For example, in the axolotl, *Fgf8*, a gene critical for limb regeneration, is situated within an expanded TAD compared with other vertebrates, which may help preserve tissue-specific transcriptional regulation despite genome expansion.^4^ Second, newly identified regulatory elements termed REX (Range EXtender; [C/T]AATTA) motifs compensate for the extended distances associated with long-range enhancers in mammals.^60^ REX motifs are predicted to bind homeobox transcription factors and are located within or near long-range enhancers, such as MFCS1/ZRS of the *Shh* gene and HS72 of the *Sall1* gene, enabling their long-range regulatory functions. We propose that these two mechanisms could act as safeguards, preserving the constrained developmental program from the disruptive effects of genome expansion. Investigating the mechanisms of gene regulation by long-range CNEs in salamander model organisms such as newts and axolotls offers a promising new research direction.

Intriguingly, in this study, the regeneration-associated *cis*-element identified in the newt MFCS1/ZRS region corresponds to one of the three REX motifs recently reported within mammalian MFCS1/ZRS, where homeobox transcription factors not only facilitate long-range promoter-enhancer interactions but also could establish a chromatin state favorable for gene reactivation during regeneration. While the activity of this functional element during regeneration remains hypothetical, future studies employing Hi-C to compare TAD in development versus regeneration, alongside chromatin immunoprecipitation sequencing and Assay for Transposase-Accessible Chromatin using sequencing (ATAC-seq) to assess chromatin states, will be indispensable for dissecting its molecular mechanism in detail. Although previous research has established the role of *Hoxc12/13* in promoting limb regeneration in *X. laevis*,^61^ elucidating the molecular mechanisms will depend on the functional identification of key *cis*-elements and their partner *trans*-factors in organ regeneration. In addition, functional genomics approaches, such as CRISPR-Cas9-mediated disruption of these candidate elements and the subsequent evaluation of their effects on organ regeneration, are anticipated to be instrumental in uncovering the detailed mechanisms underlying organ regeneration mediated by *cis-trans* interactions. *P. waltl*, a urodele amphibian with rapid sexual maturity and remarkable amenability to reverse genetics, represents a versatile and promising model organism for advancing our understanding of organ regeneration. Leveraging these tools and our incipient inbred line with genome data promises to deepen our understanding of molecular mechanisms of regeneration, fostering breakthroughs in regenerative medicine.

### Limitations of the study

The inbred newt data are still in a draft state; therefore, chromosome-level assembly using Hi-C and further curation and annotation with Iso-Seq are required for the completion of the inbred *P. waltl* genome. In addition to the wild-type data in the previous report, the inbred data will complement the wild-type data to build a reference genome for *P. waltl*. Furthermore, additional analyses of the regeneration-associated *cis*-element should be conducted to examine the hypothesis proposed in this study.

## Resource availability

### Lead contact

Should further information or requests for resources be required, these should be directed to the lead contact, who will ensure that they are fulfilled, Ken-ichi T. Suzuki (suzuk107@nibb.ac.jp).

### Materials availability

The newt line used in this study will be available from Hiroshima University via the National BioResource Project by the Ministry of Education, Culture, Sports, Science and Technology (MEXT) of Japan. However, it may require a payment and a completed materials transfer agreement (MTA).

### Data and code availability

• Data: All RNA and DNA sequencing data were deposited in DDBJ BioProject (accession no. PRJDB19556).
• Code: This paper does not report original code.
• Other: This paper does not report any additional resources.

## Acknowledgments

We are grateful to Drs. Shigehiro Kuraku and Kazuharu Arakawa for their valuable advice, which greatly facilitated the progress of this project. The illustrations of the organisms included in Figure S1 were drawn by Mr. Yujiro Kawabe. We thank Drs. Elly M. Tanaka and Akane Kawaguchi for providing the CNE80 vector and Drs. Joost M. Woltering and Axel Meyer for the *N. forsteri* Hox gene sequence. The incipient inbred *P. waltl* newts were provided by Hiroshima University Amphibian Research Center and the National BioResource Project of MEXT. Computational resources were provided by the Data Integration and Analysis Facility, National Institute for Basic Biology. Computations were partially performed on the NIG supercomputer at ROIS National Institute of Genetics. The computation was performed using Research Center for Computational Science, Okazaki, Japan (Project: NIBB, 24-IMS-C385 and 25-IMS-C354), and Chuo University Institute of Science and Engineering to A.F. This research was supported by the following funding sources: JST, 10.13039/501100003382CREST Grant Number JPMJCR2025 (K.T.S.); 10.13039/501100001691JSPS KAKENHI Grant Number JP16H06279 (PAGS); JP21H03829, JP22H04925 (PAGS); JP18K06257, JP22K06822, JP16H04794, JP19K07268, JP22H02796, JP21K20667 (K.T.S., M.T., T.T, T.H., and Y. Shibata); NIBB Collaborative Research Program (22NIBB451, 23NIBB438, 24NIBB457, 25NIBB402, 21NIBB101, 22NIBB201, 23NIBB201) to M.M. and T.H.; and Joint Research of the 10.13039/501100019770Exploratory Research Center on Life and Living Systems Grant Numbers 23-S6, 22-S3 (K.T.S). We thank Gabe Yedid, PhD, from Edanz (https://jp.edanz.com/ac), for editing a draft of this manuscript.

## Author contributions

Conceptualization, K.T.S. and T.H.; methodology, A.O., H.N., M.M., and K.T.S.; validation, M.S., M.M., and K.T.S.; formal analysis, Y.K., M.S., A.O., M.M., H.N., R.M., K.B., T.N., I.H., Y.H., A.F., T.I., Y. Sato, Z.Z., A.C., M.T., Y. Shibata, M.K., I.U., S.S., and K.T.S.; investigation, Y.K., M.S., Y.U., K.Y., Y. Suzuki, A.T., and K.T.S.; resources, N.M. and T.H.; data curation, Y.K., M.S., A.O., M.M., H.N., T.I., Y. Sato, K.Y., A.T., T.H., and K.T.S.; writing – original draft, Y.K., M.S., A.O., M.M., H.N., R.M., Y.U., T.N., I.H., Y.H., K.Y., S.S., T.H., and K.T.S.; writing – review & editing, all members; visualization, Y.K., M.S., A.O., M.M., R.M., K.B., Y.U., I.H., Y.H., A.F., T.I., Y. Sato, A.C., and K.T.S.; supervision, I.U., T.T., M.N., K.A., S.S., T.H., and K.T.S.; project administration, S.S., T.H., and K.T.S.; funding acquisition, Y. Shibata, M.K., T.T., T.H., and K.T.S.

## Declaration of interests

The authors declare no competing interests.

## Declaration of generative AI and AI-assisted technologies in the writing process

The authors acknowledge the use of ChatGPT and Grammarly for language editing support. All AI-assisted texts were reviewed and revised by the authors. The authors take full responsibility for the contents.

## STAR★Methods

### Key resources table

**Table:** 

REAGENT or RESOURCE	SOURCE	IDENTIFIER
**Critical commercial assays**		

Nanobind Tissue Big DNA Kit	Circulomics	#NB-900-701-01
BioMasher II	Nippi	#320103
SMRTbell Express Template Prep Kit 2.0	Pacific Biosciences	#100-938-900
TRIzol	Thermo Fisher Scientific	#15596026

**Chemicals, peptides, and recombinant proteins**		

Tricaine methanesulfonate (MS222)	Sigma-Aldrich	Cat#E10521
Fetal Bovine Serum	Thermo Fisher Scientific	#10437-028
RPMI 1640 Medium	Thermo Fisher Scientific	#11875-093
Antibiotic-Antimycotic	Thermo Fisher Scientific	#15240-062
mercaptoethanol	FUJIFILM Wako	#131-14572
HEPES	FUJIFILM Wako	#346-01373
Concanavalin A	Sigma-Aldrich	#C5275
LPS	Sigma-Aldrich	#L2012
PHA Reagent Grade	Thermo Fisher Scientific	#R30852701
Digoxigenin-11-dUTP	Sigma-Aldrich	#11093088910
Nick translation kit	Sigma-Aldrich	#10976776001
Anti-Digoxigenin-Rhodamine, Fab fragments	Sigma-Aldrich	#11207750910; RRID: AB_514501
Vectashield mount medium with DAPI	Vector Laboratories	#H-1200

**Deposited data**		

RNA-seq data of embryos and multi-tissue	Matsunami et al.^20^	DDBJ: PRJDB6752, PRJDB7065, PRJDB6598, and PRJDB7442
RNA-seq data of limb buds	Suzuki et al.^62^	DDBJ: PRJDB19556
TSS-seq data of hearts and embryos	This paper	DDBJ: PRJDB19556
Iso-seq data of limb blastema	Brown et al.^12^	DDBJ: PRJDB19556
*Pleurodeles waltl* (Inbred) genome assembly	This paper	DDBJ: PRJDB19556
*Pleurodeles waltl* (Inbred) genome assembly annotation	This paper	figshare: https://doi.org/10.6084/m9.figshare.29540732
*Pleurodeles waltl* (Wt) genome assembly	Brown et al.^12^	Edmond: https://doi.org/10.17617/3.90C1ND
*Homo sapiens* (*H.sa*) genome assembly		GRCh38
*Mus musculus* (*M.mu*) genome assembly		GRCm39
*Gallus gallus* (*G.ga*) genome assembly		GCA_000002315.5
*Ambystoma mexicanum* (*A.me*) genome assembly		GCA_002915635.3
*Xenopus tropicalis (X.tr)* genome assembly		GCA_000004195.4
*Protopterus annectens* (*P.an*) genome assembly		GCA_019279795.1
*Microcaecilia unicolor* (*M.un*) genome assembly		GCA_901765095.2
*Danio rerio* (*D.re*) genome assembly		GCA_000002035.4

**Software and algorithms**		

RSEM	Li et al.^63^	https://deweylab.github.io/RSEM/
STAR 2.7.9a	Dobin et al.^64^	https://github.com/alexdobin/STAR
FATE ver.2.8.0	Hikoyu Suzuki	https://github.com/Hikoyu/FATE/blob/master/fate.pl
BLAST v2.14.0+	NCBI	https://ftp.ncbi.nlm.nih.gov/blast/executables/blast+/2.14.0/
GeneWise v2.4.1	Birney et al.^65^	https://www.ebi.ac.uk/∼birney/wise2/
MAFFT v7.505	Katoh et al.^66^	https://mafft.cbrc.jp/alignment/software/linux.html
RAxML-NG v1.2.0	Kozlov et al.^67^	https://github.com/amkozlov/raxml-ng/releases
ModelTest-NG v0.1.7	Darriba et al.^68^	https://github.com/ddarriba/modeltest/releases
gVolante	Nishimura et al.^69^	https://gvolante.riken.jp/
ColabFold v1.5.5(AlphaFold2 using MMseqs2)	Mirdita et al.^70^	https://github.com/sokrypton/ColabFold
ChimeraX (version 1.7)	Goddard et al.^71^	https://www.rbvi.ucsf.edu/chimerax/
JASPAR	Rauluseviciute et al.^72^	https://jaspar.elixir.no/
BRAKER 2.1.6	Brůna et al.^73^	https://github.com/Gaius-Augustus/BRAKER
GALBA 1.0.7	Brůna et al.^74^	https://github.com/Gaius-Augustus/GALBA
TSEBRA 1.1.0	Gabriel et al.^75^	https://github.com/Gaius-Augustus/TSEBRA
cDNA_Cupcake 29.0	Tseng et al.^76^	https://github.com/Magdoll/cDNA_Cupcake
minimap2 2.24	Li et al.^77^	https://github.com/lh3/minimap2
RepeatMasker 4.1.3	Institute for Systems Biology	http://www.repeatmasker.org
RepeatModeler 2.0.3	Flynn et al.^78^	https://github.com/Dfam-consortium/RepeatModeler
TRF 4.0.9	Gary Benson	https://tandem.bu.edu/trf/trf.html
RMBlast 2.11.0	Institute for Systems Biology	https://www.repeatmasker.org/rmblast/
LTR_Retriever 2.9.0	Flynn et al.^78^	https://github.com/oushujun/LTR_retriever
RECON	Bao and Eddy^79^	http://www.repeatmasker.org
RepeatScout 1.0.6	Price et al.^80^	https://github.com/mmcco/RepeatScout
GenomeTools	https://genometools.org/contract.html	https://github.com/genometools/genometools
Ninja 0.97-cluster_only	Travis Wheeler	https://github.com/TravisWheelerLab/NINJA
CD-HIT 4.8.1	Li et al.^81^	https://sites.google.com/view/cd-hit
cTENOR	Kimura^82^	https://github.com/kim2039/cTENOR
IQ-TREE	Minh et al.^83^	http://www.iqtree.org
Cutadapt 2.5	Martin et al.^84^	https://cutadapt.readthedocs.io/en/stable/index.html#
Hifiasm v0.16.1-r375	Cheng et al.^85^	https://github.com/chhylp123/hifiasm
Miniprot	Li et al.^86^	https://github.com/lh3/miniprot
UCSC Genome Browser		https://genome.ucsc.edu/
NCBI Genome Browser		https://www.ncbi.nlm.nih.gov/gdv/
Xenbase		https://www.xenbase.org/xenbase/

### Experimental model and study participant details

In this study, we used animals of the same strain (including the F8-generation) that had undergone inbreeding from two pairs of Iberian ribbed newts (*Pleurodeles waltl*) at Tottori University and are maintained at the Amphibian Research Center, Hiroshima University.^11^ For anesthesia before the regeneration experiment and sample collection, MS-222 (Sigma, MO, USA) was used at a final concentration of 0.1%. All animal care was approved by the Institutional Animal Care and Use Committee of the National Institutes of Natural Sciences, Tottori University, and Hiroshima University.

### Method details

#### Genomic DNA extraction, library preparation, and HiFi sequencing

Genomic DNA was isolated from the F8-generation female newt liver (1∼3.5 years old) by using a Nanobind Tissue Big DNA Kit (Circulomics #NB-900-701-01) according to the manufacturer’s protocol (HBK-TIS-001). A BioMasher II (Nippi #320103) tissue homogenizer was used for grinding, and lysis was performed at 37°C to prevent nicks. Isolated DNA was then repurified with more than 10 times QBT buffer (Qiagen, Hilden, Germany), using a Qiagen Genomic-tip 20/G. The purified DNA was fragmented into approximately 15–20 kb by needle shearing using an 18G syringe or a g-tube (Covaris, MA, USA). A HiFi library was prepared sequentially with HiFi SMRTbell Libraries using SMRTbell Express Template Prep Kit 2.0 (Pacific Bioscience, CA, USA). The obtained SMRTbell libraries were sequenced with PacBio Sequel II or IIe sequencers, and highly accurate consensus (HiFi) reads data were then obtained using the ‘pb_ccs’ workflow.

#### Transgenic and knock-out newts

Transgenic and knock-out newts were produced in accordance with previously described methods.^10^^,^^11^ Axolotl CNE80 vector was provided by Drs. Akane Kawaguchi and Elly M. Tanaka, Research Institute of Molecular Pathology, Vienna, Austria.^4^ MFCS1/ZRS mutants were maintained in-house. Genotyping was performed by Sanger sequencing.

#### Bulk RNA-seq and TSS-seq

To annotate the genome and calculate TPM, bulk mRNA sequence data of embryos, multi-tissue and limb buds (stage 34 for forelimb, stage 40 for hindlimb) of *P. waltl* were obtained from DRA (PRJDB6752, PRJDB7065, PRJDB6598, and PRJDB7442) and NCBI BioProject (PRJDB15083).^20^^,^^62^ RSEM using STAR 2.7.9a was used for mapping and counting of these sequencing data.^63^^,^^64^

To detect transcriptional start sites (TSS), we performed TSS-seq. We first collected hearts (sham-operated and 1-week post-regeneration) and embryos (stages 25 and 35) of *P. waltl* followed by total RNA extraction with TRIzol (Thermo Fisher Scientific, CA, USA). Sequencing library preparation and sequencing were performed according to the standard TSS-seq protocol.^87^

#### Genome assembly and gene annotation

Adapter sequences were filtered using cutadapt v2.5.^84^ We set the maximum error rate (-e) as 0.1 and minimum overlap length (-O) as 35, and subsequently discarded trimmed HiFi-reads. We used the filtered HiFi-reads to conduct k-mer analysis with Merqury v1.3 and GenomeScope2 pipeline.^13^^,^^15^ Because the very large newt genomes include many repetitive elements, k-mer = 64 was used for analysis. We generated k-mer spectra using Meryl,^15^ and the genome size and heterozygosity were estimated on the basis of genome profiling using GenomeScope2. We also estimated maximum read depth from the output of GenomeScope2.

Filtered HiFi-reads were used for *de novo* assembly using hifiasm 0.16.1-r375 with a purge duplication level (-l) of 1,^85^ and coverage upper bound of purge duplication (-purge-max) as 60; these parameters were estimated by k-mer analysis. To evaluate the quality of genome assembly, we chose two approaches: copy numbers using k-mers and search for universal single-copy ortholog genes, as implemented in Merqury v1.3 and BUSCO,^15^^,^^88^ respectively. On the basis of k-mer spectra generated by Meryl, we estimated base-level accuracy and completeness of assembly using Merqury. We also conducted BUSCO analysis via the gVolante webtool.^69^ Assembled genome sequences were compared with Core Vertebrate Genes (CVG) from BUSCO 5.^16^

Repeat sequences in the *P. waltl* draft genome were masked using RepeatMasker 4.1.3 with a custom repeat library generated by RepeatModeler 2.0.3,^78^ resulting in 73.26% of the genomic bases masked as repeat regions. The hard-masked genome from RepeatMasker was further processed to identify and mask tandem repeats using the Tandem Repeats Finder (TRF).^89^ The following parameters were used for the TRF run: 2 7 7 80 10 50 30, with the flags -f, -d, and -m activated. After this additional masking step, the overall masked rate for the genome reached 73.60%.

Gene prediction was performed on the soft-masked genome sequence using GALBA 1.0.7,^74^ which was executed in two distinct runs with different sets of protein sequences as training data. In the first run, protein sequences were obtained from *P. waltl* RNA-seq data, which were assembled *de novo* using Trinity 2.4.0 and translated into amino acid sequences using TransDecoder v3.0.1.^90^^,^^91^ In the second run, amino acid sequences from *A. mexicanum* were used as training data. For each run, only the predicted transcripts supported by at least one training data set of any type were retained.

To annotate the full length of genes, Iso-seq data of limb blastema were obtained from the NCBI BioProject (PRJDB19556).^12^ To integrate Iso-seq data, we followed the long-read protocol of BRAKER2.^73^^,^^92^ Briefly, Iso-seq reads were aligned to the soft-masked genome using minimap2,^93^ followed by folding redundant isoforms using Cupcake.^76^ Subsequently, GeneMarkS-T was employed to predict the protein-coding regions within these transcripts,^94^ and a GTF file was generated using the gmst2globalCoords.py script provided by BRAKER.^73^ In the final step, transcript sequences containing more than 25% repetitive elements were filtered out, resulting in a final set of 42,712 gene models and 48,801 transcripts.

All three GTF files (two generated by GALBA and one based on Iso-seq data) were merged using TSEBRA v1.1.0.^75^ In this process, the --filter_short option was added, and the configuration was set to P:3, E:0.15, C:15, M:0.5, L:5, is:1, stos:1, stas:2, e_1:0.1, e_2:1, e_3:1, e_4:300, e_5:50, e_6:20. In particular, the value for 'L' was increased to maximize the use of long-read data. GTF files are available from figshare (10.6084/m9.figshare.29540732).

#### Transposable elements annotation and analysis

Transposable elements and repetitive sequences were annotated using RepeatModeler 2.0.3 with RepeatMasker 4.1.3 and RMBlast 2.11.0+ (http://www.repeatmasker.org),^78^ TRF 4.09,^89^ RECON 1.08,^79^ RepeatScout 1.0.6,^80^ GenomeTools 1.6.3,^95^ LTR_Retriever 2.9.0,^96^ Ninja 0.97-cluster_only,^97^ MAFFT 7.505,^66^ and CD-HIT 4.8.1.^81^ RepeatModeler was executed with the -LTRStruct option. cTENOR (https://github.com/kim2039/cTENOR)^82^ with DeepTE and TransposonUltimate RFSB was used for classification of ‘Unknown’ in the output library of RepeatModeler.^98^^,^^99^ The locations of transposable elements were identified using RepeatMasker 4.1.3 with RMBlast 2.11.0+. To identify longer tandem repeat sequences, the “-l 10” option was added in the TRF.pm file. The data for TE landscape and TE proportion analyses were generated by the calcDivergenceFromAlign.pl script in RepeatMasker. BLAST, MAFFT, and LAST were used to search for microsatellites already reported in newts (GenBank: AB238602.1).^100^

On the basis of the GFF files of genomes for *P. waltl*, axolotl, lungfish, and human, intron lengths were extracted and sorted in order of length. The top 5% of introns were extracted and duplicates were removed. The obtained lists were analyzed for biological processes using Webgestalt for over-representation analysis.^101^

#### Cell culture, chromosome preparation, and FISH

For collection of lymphocytes, the spleen of a female individual was crushed between two sterilized glass slides in 6 ml of wash medium, 60% RPMI 1640 medium supplemented with 10% FBS and 1% antibiotic–antimycotic solution (all from Thermo Fisher Scientific GIBCO). After centrifugation at 1,000 rpm for 5 min at room temperature, the lymphocytes were suspended in 60% RPMI 1640 medium supplemented with 10% FBS, 1% antibiotic–antimycotic solution, 25 mM mercaptoethanol, 20 mM HEPES, and mitogens such as 6 mg/ml Con A (type IV-S) (Sigma-Aldrich, MO, USA), 20 mg/ml LPS (Sigma-Aldrich), and 18 mg/ml PHA (HA15) (Thermo Fisher Scientific) in plastic bottles for 5–7 days at 22°C in a humidified atmosphere. Following harvesting, the cultured lymphocytes were collected after colchicine treatment (200 mg/ml) for 48 h, subjected to hypotonic treatment in 0.075 M KCl for 20–40 min and fixed in methanol/acetic acid (3:1). The cell suspension was placed on a glass slide and air-dried, and the slides were kept at −80°C until use.

Fluorescence *in situ* hybridization (FISH) was performed as described previously.^102^^,^^103^ To determine the location of Pwa-ST-Bam, we used an isolated 311 bp satellite DNA of Pwa-ST-Bam as a FISH probe. For FISH of the 18S–28S rRNA genes, we used pHr21Ab (5.8 kb for the 5’ portion) and pHr14E3 (7.3 kb for the 3’ portion) fragments, which were provided by the National Institutes of Biomedical Innovation, Health and Nutrition, Osaka, as in our previous studies.^104^^,^^105^ These fragments are derived from the human 45S pre-ribosomal RNA gene (*RNA45S*), which encodes a precursor RNA for 18S, 5.8S and 28S rRNAs. The DNA fragments were labeled with digoxigenin (DIG) -11-dUTP using a nick translation kit (Roche Diagnostics, Rotkreuz, Switzerland) following the manufacturer’s instructions. After washing, the slides were incubated under parafilm with rhodamine-conjugated anti-DIG Fab fragments (Roche Diagnostics) and mounted using Vectashield mount medium with DAPI (Vector Laboratories, CA, USA).

#### Phylogenetic analysis

Amino acid sequences were aligned using MAFFT.^100^ Maximum likelihood phylogenetic trees were inferred with RAxML^68^^,^^106^ using the PROTGAMMAJTT model with 1000 bootstrap replicates and with IQ-TREE.^83^ Trees were visualized with FigTree (http://tree.bio.ed.ac.uk/software/figtree/). The set of amino acid sequences of *N. forsteri* Hox genes was provided by J. Woltering and A. Meyer.^5^^,^^107^

#### Protein structures predicted by AlphaFold2

Protein structure models were generated by ColabFold v1.5.5 (AlphaFold2 using MMseqs2),^70^^,^^108^ with all running options at default values. All structure figures were generated using ChimeraX (version 1.7rc 202311290355).^71^^,^^109^

#### Searching for transcription factor binding sites

For JASPAR (https://jaspar.elixir.no/) score calculation, both wild-type and mutant genome sequences of MFCS1/ZRS gRNA sites were submitted to JASPAR Scan.^72^ All transcription factors in the human CORE dataset, except those with binding motifs longer than 20 bases, were used for motif analysis. The relative threshold score was set to 80% (default).

#### Genome browsing

To annotate the gene structures within the *P. waltl* genome, we used the amino acid sequences from the proteomes of *Homo sapiens*, *Xenopus laevis*, and *Xenopus tropicalis*. These sequences were obtained from the UniProt Reference Proteomes database (https://www.uniprot.org/proteomes) with the respective Proteome IDs UP000005640, UP000186698, and UP000008143. We employed the software miniprot,^86^ using default settings, to map these sequences to the *P. waltl* genome. Additionally, an earlier version of gene models (PLEWA04_ORF.cds.fa), constructed from *de novo* assembly of RNA-seq data from our previous transcriptome study,^20^ was mapped to the genome assembly using minimap2 with the parameters: “-ax splice:hq”.^77^ Mapping results were visualized using JBrowse2 Web 2.7.0 (https://d1m10bmlxkjccx.cloudfront.net/jb/PleWal_pwal1_j231006dev/index.html).^110^ We manually compared the locus and CNEs described in this study across selected species without genome-wide synteny or CNE analysis to confirm orthology and evolutionary conservation.

### Quantification and statistical analysis

Where not explicitly stated in the method details section, all quantitative and statistical analyses were performed using the default parameters of the respective software packages.

## References

[1] Spemann H., Mangold H. (1924). über Induktion von Embryonalanlagen durch Implantation artfremder Organisatoren. Archiv f. mikr. Anat. u. Entwicklungsmechanik.

[2] Joven A., Elewa A., Simon A. (2019). Model systems for regeneration: salamanders. Development.

[3] Tanaka E.M. (2016). The molecular and cellular choreography of appendage regeneration. Cell.

[4] Schloissnig S., Kawaguchi A., Nowoshilow S., Falcon F., Otsuki L., Tardivo P., Timoshevskaya N., Keinath M.C., Smith J.J., Voss S.R., Tanaka E.M. (2021). The giant axolotl genome uncovers the evolution, scaling, and transcriptional control of complex gene loci. Proc. Natl. Acad. Sci. USA.

[5] Meyer A., Schloissnig S., Franchini P., Du K., Woltering J.M., Irisarri I., Wong W.Y., Nowoshilow S., Kneitz S., Kawaguchi A. (2021). Giant lungfish genome elucidates the conquest of land by vertebrates. Nature.

[6] Wang K., Wang J., Zhu C., Yang L., Ren Y., Ruan J., Fan G., Hu J., Xu W., Bi X. (2021). African lungfish genome sheds light on the vertebrate water-to-land transition. Cell.

[7] Schartl M., Woltering J.M., Irisarri I., Du K., Kneitz S., Pippel M., Brown T., Franchini P., Li J., Li M. (2024). The genomes of all lungfish inform on genome expansion and tetrapod evolution. Nature.

[8] Nowoshilow S., Schloissnig S., Fei J.-F., Dahl A., Pang A.W.C., Pippel M., Winkler S., Hastie A.R., Young G., Roscito J.G. (2018). The axolotl genome and the evolution of key tissue formation regulators. Nature.

[9] Elewa A., Wang H., Talavera-López C., Joven A., Brito G., Kumar A., Hameed L.S., Penrad-Mobayed M., Yao Z., Zamani N. (2017). Reading and editing the Pleurodeles waltl genome reveals novel features of tetrapod regeneration. Nat. Commun..

[10] Suzuki M., Hayashi T., Inoue T., Agata K., Hirayama M., Suzuki M., Shigenobu S., Takeuchi T., Yamamoto T., Suzuki K.-I.T. (2018). Cas9 ribonucleoprotein complex allows direct and rapid analysis of coding and noncoding regions of target genes in Pleurodeles waltl development and regeneration. Dev. Biol..

[11] Hayashi T., Yokotani N., Tane S., Matsumoto A., Myouga A., Okamoto M., Takeuchi T. (2013). Molecular genetic system for regenerative studies using newts. Dev. Growth Differ..

[12] Brown T., Mishra K., Elewa A., Iarovenko S., Subramanian E., Araus A.J., Petzold A., Fromm B., Friedländer M.R., Rikk L. (2025). Chromosome-scale genome assembly reveals how repeat elements shape non-coding RNA landscapes active during newt limb regeneration. Cell Genom..

[13] Ranallo-Benavidez T.R., Jaron K.S., Schatz M.C. (2020). GenomeScope 2.0 and Smudgeplot for reference-free profiling of polyploid genomes. Nat. Commun..

[14] Manni M., Berkeley M.R., Seppey M., Simão F.A., Zdobnov E.M. (2021). BUSCO update: novel and streamlined workflows along with broader and deeper phylogenetic coverage for scoring of eukaryotic, prokaryotic, and viral genomes. Mol. Biol. Evol..

[15] Rhie A., Walenz B.P., Koren S., Phillippy A.M. (2020). Merqury: reference-free quality, completeness, and phasing assessment for genome assemblies. Genome Biol..

[16] Hara Y., Tatsumi K., Yoshida M., Kajikawa E., Kiyonari H., Kuraku S. (2015). Optimizing and benchmarking de novo transcriptome sequencing: from library preparation to assembly evaluation. BMC Genom..

[17] Murakami T., Maki N., Nishida-Umehara C., Matsuda Y., Agata K. (2007). Establishment of high-resolution FISH mapping system and its application for molecular cytogenetic characterization of chromosomes in newt, Cynops pyrrhogaster (Urodela, Amphibia). Chromosome Res..

[18] Duboule D. (2007). The rise and fall of Hox gene clusters. Development.

[19] Liang D., Wu R., Geng J., Wang C., Zhang P. (2011). A general scenario of Hox gene inventory variation among major sarcopterygian lineages. BMC Evol. Biol..

[20] Matsunami M., Suzuki M., Haramoto Y., Fukui A., Inoue T., Yamaguchi K., Uchiyama I., Mori K., Tashiro K., Ito Y. (2019). A comprehensive reference transcriptome resource for the Iberian ribbed newt Pleurodeles waltl, an emerging model for developmental and regeneration biology. DNA Res..

[21] Rudnicki M.A., Schnegelsberg P.N., Stead R.H., Braun T., Arnold H.H., Jaenisch R. (1993). MyoD or Myf-5 is required for the formation of skeletal muscle. Cell.

[22] Simakov O., Kawashima T., Marlétaz F., Jenkins J., Koyanagi R., Mitros T., Hisata K., Bredeson J., Shoguchi E., Gyoja F. (2015). Hemichordate genomes and deuterostome origins. Nature.

[23] Mio C., Baldan F., Damante G. (2023). NK2 homeobox gene cluster: Functions and roles in human diseases. Genes Dis..

[24] Amemiya Y., Sogabe Y., Nozaki M., Takahashi A., Kawauchi H. (1999). Somatolactin in the white sturgeon and African lungfish and its evolutionary significance. Gen. Comp. Endocrinol..

[25] Kaneko T., Jeon K.W. (1996). International Review of Cytology.

[26] Kikuyama S., Toyoda F., Ohmiya Y., Matsuda K., Tanaka S., Hayashi H. (1995). Sodefrin: a female-attracting peptide pheromone in newt cloacal glands. Science.

[27] Toyoda F., Kikuyama S. (2000). Hormonal influence on the olfactory response to a female-attracting pheromone, sodefrin, in the newt, Cynops pyrrhogaster. Comp. Biochem. Physiol. B Biochem. Mol. Biol..

[28] Van Bocxlaer I., Maex M., Treer D., Janssenswillen S., Janssens R., Vandebergh W., Proost P., Bossuyt F. (2016). Beyond sodefrin: evidence for a multi-component pheromone system in the model newt Cynops pyrrhogaster (Salamandridae). Sci. Rep..

[29] Nakada T., Ishizuka Y., Iwata T., Toyoda F., Kato T., Conlon J.M., Kikuyama S. (2007). Evidence for processing enzymes in the abdominal gland of the newt, Cynops pyrrhogaster, that generate sodefrin from its biosynthetic precursor. Zoolog. Sci..

[30] Smemo S., Campos L.C., Moskowitz I.P., Krieger J.E., Pereira A.C., Nobrega M.A. (2012). Regulatory variation in a TBX5 enhancer leads to isolated congenital heart disease. Hum. Mol. Genet..

[31] Tatsumi N., Kobayashi R., Yano T., Noda M., Fujimura K., Okada N., Okabe M. (2016). Molecular developmental mechanism in polypterid fish provides insight into the origin of vertebrate lungs. Sci. Rep..

[32] Sagai T., Hosoya M., Mizushina Y., Tamura M., Shiroishi T. (2005). Elimination of a long-range cis-regulatory module causes complete loss of limb-specific Shh expression and truncation of the mouse limb. Development.

[33] Kvon E.Z., Kamneva O.K., Melo U.S., Barozzi I., Osterwalder M., Mannion B.J., Tissières V., Pickle C.S., Plajzer-Frick I., Lee E.A. (2016). Progressive loss of function in a limb enhancer during snake evolution. Cell.

[34] Sun C., Shepard D.B., Chong R.A., López Arriaza J., Hall K., Castoe T.A., Feschotte C., Pollock D.D., Mueller R.L. (2012). LTR retrotransposons contribute to genomic gigantism in plethodontid salamanders. Genome Biol. Evol..

[35] Sun C., López Arriaza J.R., Mueller R.L. (2012). Slow DNA loss in the gigantic genomes of salamanders. Genome Biol. Evol..

[36] Skipper K.A., Andersen P.R., Sharma N., Mikkelsen J.G. (2013). DNA transposon-based gene vehicles - scenes from an evolutionary drive. J. Biomed. Sci..

[37] Kumar S., Suleski M., Craig J.M., Kasprowicz A.E., Sanderford M., Li M., Stecher G., Hedges S.B. (2022). TimeTree 5: An expanded resource for species divergence times. Mol. Biol. Evol..

[38] Winnier G., Blessing M., Labosky P.A., Hogan B.L. (1995). Bone morphogenetic protein-4 is required for mesoderm formation and patterning in the mouse. Genes Dev..

[39] Vincent E., Villiard E., Sader F., Dhakal S., Kwok B.H., Roy S. (2020). BMP signaling is essential for sustaining proximo-distal progression in regenerating axolotl limbs. Development.

[40] Megeney L.A., Kablar B., Garrett K., Anderson J.E., Rudnicki M.A. (1996). MyoD is required for myogenic stem cell function in adult skeletal muscle. Genes Dev..

[41] Gayraud-Morel B., Chrétien F., Flamant P., Gomès D., Zammit P.S., Tajbakhsh S. (2007). A role for the myogenic determination gene Myf5 in adult regenerative myogenesis. Dev. Biol..

[42] Hecker L., Jagirdar R., Jin T., Thannickal V.J. (2011). Reversible differentiation of myofibroblasts by MyoD. Exp. Cell Res..

[43] Beccari L., Yakushiji-Kaminatsui N., Woltering J.M., Necsulea A., Lonfat N., Rodríguez-Carballo E., Mascrez B., Yamamoto S., Kuroiwa A., Duboule D. (2016). A role for HOX13 proteins in the regulatory switch between TADs at the HoxD locus. Genes Dev..

[44] Lopez-Delisle L., Zakany J., Bochaton C., Osteil P., Mayran A., Darbellay F., Mascrez B., Rekaik H., Duboule D. (2024). CTCF-dependent insulation of Hoxb13 and the heterochronic control of tail length. Proc. Natl. Acad. Sci. USA.

[45] Fromental-Ramain C., Warot X., Messadecq N., LeMeur M., Dollé P., Chambon P. (1996). Hoxa-13 and Hoxd-13 play a crucial role in the patterning of the limb autopod. Development.

[46] Takeuchi T., Matsubara H., Minamitani F., Satoh Y., Tozawa S., Moriyama T., Maruyama K., Suzuki K.-I.T., Shigenobu S., Inoue T. (2022). Newt Hoxa13 has an essential and predominant role in digit formation during development and regeneration. Development.

[47] Watanabe M., Yasuoka Y., Mawaribuchi S., Kuretani A., Ito M., Kondo M., Ochi H., Ogino H., Fukui A., Taira M., Kinoshita T. (2017). Conservatism and variability of gene expression profiles among homeologous transcription factors in Xenopus laevis. Dev. Biol..

[48] Lukas P., Schmidt J., Olsson L. (2019). Knockdown of zax in Xenopus laevis leads to craniofacial malformations and the absence of the intramandibular joint. VZ.

[49] Square T., Jandzik D., Cattell M., Coe A., Doherty J., Medeiros D.M. (2015). A gene expression map of the larval Xenopus laevis head reveals developmental changes underlying the evolution of new skeletal elements. Dev. Biol..

[50] Fukamachi S., Meyer A. (2007). Evolution of receptors for growth hormone and somatolactin in fish and land vertebrates: lessons from the lungfish and sturgeon orthologues. J. Mol. Evol..

[51] Fukamachi S., Sugimoto M., Mitani H., Shima A. (2004). Somatolactin selectively regulates proliferation and morphogenesis of neural-crest derived pigment cells in medaka. Proc. Natl. Acad. Sci. USA.

[52] Fukamachi S., Yada T., Mitani H. (2005). Medaka receptors for somatolactin and growth hormone: phylogenetic paradox among fish growth hormone receptors. Genetics.

[53] Fukamachi S., Wakamatsu Y., Mitani H. (2006). Medaka double mutants for color interfere and leucophore free: characterization of the xanthophore-somatolactin relationship using the leucophore free gene. Dev. Genes Evol..

[54] Fukamachi S., Kinoshita M., Aizawa K., Oda S., Meyer A., Mitani H. (2009). Dual control by a single gene of secondary sexual characters and mating preferences in medaka. BMC Biol..

[55] Lüdtke D.U., Foerster K. (2018). Choosy males court both large, colourful females and less colourful but responsive females for longer. Anim. Behav..

[56] Bossuyt F., Schulte L.M., Maex M., Janssenswillen S., Novikova P.Y., Biju S.D., Van de Peer Y., Matthijs S., Roelants K., Martel A., Van Bocxlaer I. (2019). Multiple independent recruitment of sodefrin precursor-like factors in anuran sexually dimorphic glands. Mol. Biol. Evol..

[57] Bossuyt F., Maex M., Treer D., Schulte L.M., Van Bocxlaer I., Janssenswillen S., Buesching C. (2019). Chemical Signals in Vertebrates 14.

[58] Wilburn D.B., Kunkel C.L., Feldhoff R.C., Feldhoff P.W., Searle B.C. (2022). Recurrent co-option and recombination of cytokine and three finger proteins in multiple reproductive tissues throughout salamander evolution. Front. Cell Dev. Biol..

[59] Kimoto H., Haga S., Sato K., Touhara K. (2005). Sex-specific peptides from exocrine glands stimulate mouse vomeronasal sensory neurons. Nature.

[60] Bower G., Hollingsworth E.W., Jacinto S.H., Alcantara J.A., Clock B., Cao K., Liu M., Dziulko A., Alcaina-Caro A., Xu Q. (2025). Range extender mediates long-distance enhancer activity. Nature.

[61] Kawasumi-Kita A., Lee S.-W., Ohtsuka D., Niimi K., Asakura Y., Kitajima K., Sakane Y., Tamura K., Ochi H., Suzuki K.-I.T., Morishita Y. (2024). hoxc12/c13 as key regulators for rebooting the developmental program in Xenopus limb regeneration. Nat. Commun..

[62] Suzuki M., Okumura A., Chihara A., Shibata Y., Endo T., Teramoto M., Agata K., Bronner M.E., Suzuki K.-I.T. (2024). Fgf10 mutant newts regenerate normal hindlimbs despite severe developmental defects. Proc. Natl. Acad. Sci. USA.

[63] Li B., Dewey C.N. (2011). RSEM: accurate transcript quantification from RNA-Seq data with or without a reference genome. BMC Bioinf..

[64] Dobin A., Davis C.A., Schlesinger F., Drenkow J., Zaleski C., Jha S., Batut P., Chaisson M., Gingeras T.R. (2013). STAR: ultrafast universal RNA-seq aligner. Bioinformatics.

[65] Birney E., Clamp M., Durbin R. (2004). GeneWise and Genomewise. Genome Res..

[66] Katoh K., Standley D.M. (2013). MAFFT multiple sequence alignment software version 7: improvements in performance and usability. Mol. Biol. Evol..

[67] Kozlov A.M., Darriba D., Flouri T., Morel B., Stamatakis A. (2019). RAxML-NG: a fast, scalable and user-friendly tool for maximum likelihood phylogenetic inference. Bioinformatics.

[68] Darriba D., Posada D., Kozlov A.M., Stamatakis A., Morel B., Flouri T. (2020). ModelTest-NG: A new and scalable tool for the selection of DNA and protein evolutionary models. Mol. Biol. Evol..

[69] Nishimura O., Hara Y., Kuraku S. (2017). gVolante for standardizing completeness assessment of genome and transcriptome assemblies. Bioinformatics.

[70] Mirdita M., Schütze K., Moriwaki Y., Heo L., Ovchinnikov S., Steinegger M. (2022). ColabFold: making protein folding accessible to all. Nat. Methods.

[71] Goddard T.D., Huang C.C., Meng E.C., Pettersen E.F., Couch G.S., Morris J.H., Ferrin T.E. (2018). UCSF ChimeraX: Meeting modern challenges in visualization and analysis. Protein Sci..

[72] Rauluseviciute I., Riudavets-Puig R., Blanc-Mathieu R., Castro-Mondragon J.A., Ferenc K., Kumar V., Lemma R.B., Lucas J., Chèneby J., Baranasic D. (2024). JASPAR 2024: 20th anniversary of the open-access database of transcription factor binding profiles. Nucleic Acids Res..

[73] Brůna T., Hoff K.J., Lomsadze A., Stanke M., Borodovsky M. (2021). BRAKER2: automatic eukaryotic genome annotation with GeneMark-EP+ and AUGUSTUS supported by a protein database. NAR Genom. Bioinform..

[74] Brůna T., Li H., Guhlin J., Honsel D., Herbold S., Stanke M., Nenasheva N., Ebel M., Gabriel L., Hoff K.J. (2023). Galba: genome annotation with miniprot and AUGUSTUS. BMC Bioinf..

[75] Gabriel L., Hoff K.J., Brůna T., Borodovsky M., Stanke M. (2021). TSEBRA: transcript selector for BRAKER. BMC Bioinf..

[76] Tseng E. cDNA_Cupcake: Miscellaneous collection of Python and R scripts for processing Iso-Seq data (Github). https://github.com/Magdoll/cDNA_Cupcake.

[77] Li H. (2018). Minimap2: pairwise alignment for nucleotide sequences. Bioinformatics.

[78] Flynn J.M., Hubley R., Goubert C., Rosen J., Clark A.G., Feschotte C., Smit A.F. (2020). RepeatModeler2 for automated genomic discovery of transposable element families. Proc. Natl. Acad. Sci. USA.

[79] Bao Z., Eddy S.R. (2002). Automated de novo identification of repeat sequence families in sequenced genomes. Genome Res..

[80] Price A.L., Jones N.C., Pevzner P.A. (2005). De novo identification of repeat families in large genomes. Bioinformatics.

[81] Li W., Godzik A. (2006). Cd-hit: a fast program for clustering and comparing large sets of protein or nucleotide sequences. Bioinformatics.

[82] Kimura Y. (2022). https://github.com/kim2039/cTENOR.

[83] Minh B.Q., Schmidt H.A., Chernomor O., Schrempf D., Woodhams M.D., von Haeseler A., Lanfear R. (2020). IQ-TREE 2: New models and efficient methods for phylogenetic inference in the genomic era. Mol. Biol. Evol..

[84] Martin M. (2011). Cutadapt removes adapter sequences from high-throughput sequencing reads. EMBnet. J..

[85] Cheng H., Concepcion G.T., Feng X., Zhang H., Li H. (2021). Haplotype-resolved de novo assembly using phased assembly graphs with hifiasm. Nat. Methods.

[86] Li H. (2023). Protein-to-genome alignment with miniprot. Bioinformatics.

[87] Tsuchihara K., Suzuki Y., Wakaguri H., Irie T., Tanimoto K., Hashimoto S.-I., Matsushima K., Mizushima-Sugano J., Yamashita R., Nakai K. (2009). Massive transcriptional start site analysis of human genes in hypoxia cells. Nucleic Acids Res..

[88] Simão F.A., Waterhouse R.M., Ioannidis P., Kriventseva E.V., Zdobnov E.M. (2015). BUSCO: assessing genome assembly and annotation completeness with single-copy orthologs. Bioinformatics.

[89] Benson G. (1999). Tandem repeats finder: a program to analyze DNA sequences. Nucleic Acids Res..

[90] Grabherr M.G., Haas B.J., Yassour M., Levin J.Z., Thompson D.A., Amit I., Adiconis X., Fan L., Raychowdhury R., Zeng Q. (2011). Full-length transcriptome assembly from RNA-Seq data without a reference genome. Nat. Biotechnol..

[91] Haas B.J. TransDecoder: TransDecoder source (Github). https://github.com/TransDecoder/TransDecoder.

[92] docs/long_reads/long_read_protocol.Md at Long_reads · Gaius-Augustus/BRAKER (Github). https://github.com/Gaius-Augustus/BRAKER/blob/long_reads/docs/long_reads/long_read_protocol.md.

[93] Li H. (2021). New strategies to improve minimap2 alignment accuracy. Bioinformatics.

[94] Besemer J., Lomsadze A., Borodovsky M. (2001). GeneMarkS: a self-training method for prediction of gene starts in microbial genomes. Implications for finding sequence motifs in regulatory regions. Nucleic Acids Res..

[95] Gremme G., Steinbiss S., Kurtz S. (2013). GenomeTools: a comprehensive software library for efficient processing of structured genome annotations. IEEE/ACM Trans. Comput. Biol. Bioinform..

[96] Ou S., Jiang N. (2018). LTR_retriever: a highly accurate and sensitive program for identification of long terminal repeat retrotransposons. Plant Physiol..

[97] Wheeler T., Soares M.W.D., Lesica G. (2020).

[98] Yan H., Bombarely A., Li S. (2020). DeepTE: a computational method for de novo classification of transposons with convolutional neural network. Bioinformatics.

[99] Riehl K., Riccio C., Miska E.A., Hemberg M. (2022). TransposonUltimate: software for transposon classification, annotation and detection. Nucleic Acids Res..

[100] Katoh K., Rozewicki J., Yamada K.D. (2019). MAFFT online service: multiple sequence alignment, interactive sequence choice and visualization. Brief. Bioinform..

[101] Liao Y., Wang J., Jaehnig E.J., Shi Z., Zhang B. (2019). WebGestalt 2019: gene set analysis toolkit with revamped UIs and APIs. Nucleic Acids Res..

[102] Matsuda Y., Chapman V.M. (1995). Application of fluorescence in situ hybridization in genome analysis of the mouse. Electrophoresis.

[103] Uno Y., Nishida C., Tarui H., Ishishita S., Takagi C., Nishimura O., Ishijima J., Ota H., Kosaka A., Matsubara K. (2012). Inference of the protokaryotypes of amniotes and tetrapods and the evolutionary processes of microchromosomes from comparative gene mapping. PLoS One.

[104] Session A.M., Uno Y., Kwon T., Chapman J.A., Toyoda A., Takahashi S., Fukui A., Hikosaka A., Suzuki A., Kondo M. (2016). Genome evolution in the allotetraploid frog Xenopus laevis. Nature.

[105] Uno Y., Nozu R., Kiyatake I., Higashiguchi N., Sodeyama S., Murakumo K., Sato K., Kuraku S. (2020). Cell culture-based karyotyping of orectolobiform sharks for chromosome-scale genome analysis. Commun. Biol..

[106] Stamatakis A. (2014). RAxML version 8: a tool for phylogenetic analysis and post-analysis of large phylogenies. Bioinformatics.

[107] Woltering J.M., Irisarri I., Ericsson R., Joss J.M.P., Sordino P., Meyer A. (2020). Sarcopterygian fin ontogeny elucidates the origin of hands with digits. Sci. Adv..

[108] Jumper J., Evans R., Pritzel A., Green T., Figurnov M., Ronneberger O., Tunyasuvunakool K., Bates R., Žídek A., Potapenko A. (2021). Highly accurate protein structure prediction with AlphaFold. Nature.

[109] Meng E.C., Goddard T.D., Pettersen E.F., Couch G.S., Pearson Z.J., Morris J.H., Ferrin T.E. (2023). UCSF ChimeraX: Tools for structure building and analysis. Protein Sci..

[110] Diesh C., Stevens G.J., Xie P., De Jesus Martinez T., Hershberg E.A., Leung A., Guo E., Dider S., Zhang J., Bridge C. (2023). JBrowse 2: a modular genome browser with views of synteny and structural variation. Genome Biol..

