# Supplementary material for: The inbred newt genome unveils molecular mechanisms of behavior, development, and regeneration in urodele amphibians
Source: iScience. 2025 Sep 9;28(10):113535. doi: 10.1016/j.isci.2025.113535 (PMC12506533; doi:10.1016/j.isci.2025.113535)
Supplement: Document S1. Figures S1–S22 and Tables S1–S7 [file mmc1.pdf]

iScience, Volume *28*

Supplemental information

The inbred newt genome unveils molecular mechanisms of behavior, development, and regeneration in urodele amphibians

Yuki Kimura, Miyuki Suzuki, Akinori Okumura, Masatoshi Matsunami, Hiroyo Nishide, Rima Mizuno, Kazuto Bou, Yoshinobu Uno, Tomoaki Nakada, Itaru Hasunuma, Yoshikazu Haramoto, Akimasa Fukui, Takeshi Inoue, Yuki Sato, Katsushi Yamaguchi, Zicong Zhang, Akane Chihara, Mai Takehara, Yuki Shibata, Masaaki Kitada, Nerea Moreno, Ikuo Uchiyama, Yutaka Suzuki, Takashi Takeuchi, Masato Nikaido, Kiyokazu Agata, Atsushi Toyoda, Shuji Shigenobu, Toshinori Hayashi, and Ken-ichi T. Suzuki

Supplementary Tables

Table S1. Assembly statistics and BUSCO assessment of the incipient inbred *Pleurodeles waltl*.

Table S2. *OR* numbers in vertebrates

Table S3. *TAAR* numbers in vertebrates

Table S4. *V1R* numbers in vertebrates

Table S5. *T2R* numbers in vertebrates

Table S6. *V2R* numbers in vertebrates

Table S7. *T1R* numbers in vertebrates

Table S1. Assembly statistics and BUSCO assessment of the outbred *Pleurodeles waltl*.

**Genome assembly**	**Number of sequences**	**Total length (bp)**	**N50 (bp)**	**N90 (bp)**	**Longest (bp)**	**%Gaps**
Scaffold	2,679	21,934,601,490	60,529,522	11,182,743	348,366,668	0

**BUSCO assessment**	**Total**	**Complete**	**Complete and single copy**	**Complete and duplicated**	**Fragmented**	**Missing**
CVG	233	224 (96.1%)	214 (91.8%)	10 (4.3%)	6 (2.6%)	3 (1.3%)
Vertebrata	3,354	3,051 (91.0%)	2,931 (87.4%)	120 (3.6%)	191 (5.7%)	112 (3.3%)

Table S2. The gene numbers of Olfactory receptors (OR).

		Type1									Type2					Total
Assembly ID	Species name	α	β	γ	γ-like	δ	coelacanth-spe	ε	ζ	type1-like	η	θ	κ	λ	type2-like	
ASM1683550v1	Polypterus_senegalus	28	10	52	117	69	0	21	275	0	22	1	3	2	3	603
LatCha1	Latimeria_chalumnae	6	0	15	2	71	3	0	44	1	17	0	3	0	2	164
PAN1.0	Protopterus_annectens	87	1	34	69	55	0	0	2	0	4	0	3	1	1	257
neoFor_v3.1	Neoceratodus_forsteri	40	1	39	35	55	0	0	2	0	1	2	3	1	1	180
aRhiBiv1.1	Rhinatrema_bivittatum	123	4	1073	0	0	0	0	0	0	1	0	5	0	0	1206
UCB_Xtro_10.0	Xenopus_tropicalis	13	14	616	0	31	0	17	0	0	11	0	1	0	0	703
AmbMex60DD	Ambystoma_mexicanum	105	0	751	0	0	0	0	0	0	6	0	6	3	1	872
"This study"	Pleurodeles_waltl	171	0	916	0	2	0	0	0	1	24	1	9	0	1	1125
ASM2665232v1	Pleurodeles_waltl	173	0	880	0	2	0	0	0	1	24	1	10	0	1	1092

Table S3. The gene numbers of trace amine-associated receptors (TAAR).

Assembly ID	Species name	TAARlike1	TAARlike2	class1	class2				class3	Total
					mammal2-4like	taar12	mammal5-9like	taar13		
ASM1683550v1	Polypterus_senegalus	1	0	39	0	3	27	1	243	314
LatCha1	Latimeria_chalumnae	1	1	3	0	2	17	5	0	29
PAN1.0	Protopterus_annectens	0	0	2	18	13	27	11	0	71
neoFor_v3.1	Neoceratodus_forsteri	1	0	1	7	0	15	11	0	35
aRhiBiv1.1	Rhinatrema_bivittatum	0	0	3	7	0	1	0	0	11
UCB_Xtro_10.0	Xenopus_tropicalis	1	0	1	4	0	0	0	0	6
AmbMex60DD	Ambystoma_mexicanum	2	0	2	4	0	2	0	0	10
"This study"	Pleurodeles_waltl	1	0	2	6	0	1	0	0	10
ASM2665232v1	Pleurodeles_waltl	1	0	2	6	0	1	0	0	10

Table S4. The gene numbers of vomeronasal receptor 1 genes (V1R).

Assembly ID	Species name	V1R1	V1R2	V1R3	V1R4	V1R5	V1R6	ancV1R	Total
ASM1683550v1	Polypterus_senegalus	0	45	1	1	0	1	1	49
LatCha1	Latimeria_chalumnae	1	15	0	0	0	1	1	18
PAN1.0	Protopterus_annectens	0	158	0	0	0	2	1	161
neoFor_v3.1	Neoceratodus_forsteri	0	80	0	0	0	6	0	86
aRhiBiv1.1	Rhinatrema_bivittatum	0	14	0	0	0	0	1	15
UCB_Xtro_10.0	Xenopus_tropicalis	0	20	0	0	0	0	1	21
AmbMex60DD	Ambystoma_mexicanum	1	33	0	0	0	1	1	36
"This study"	Pleurodeles_waltl	1	23	0	0	0	0	1	25
ASM2665232v1	Pleurodeles_waltl	1	24	0	0	0	0	1	26

Table S5. The gene numbers of taste receptor type 1 genes (T2R).

Assembly ID	Species name	Total
ASM1683550v1	Polypterus_senegalus	16
LatCha1	Latimeria_chalumnae	69
PAN1.0	Protopterus_annectens	12
neoFor_v3.1	Neoceratodus_forsteri	27
aRhiBiv1.1	Rhinatrema_bivittatum	16
UCB_Xtro_10.0	Xenopus_tropicalis	49
AmbMex60DD	Ambystoma_mexicanum	70
"This study"	Pleurodeles_waltl	69
ASM2665232v1	Pleurodeles_waltl	69

Table S6. The gene numbers of vomeronasal receptor 2 genes (V2R).

Assembly ID	Species name	V2R2	ancV2R	f-V2R	t-V2R	Total
ASM1683550v1	Polypterus_senegalus	1	1	172	4	178
LatCha1	Latimeria_chalumnae	1	0	13	75	89
PAN1.0	Protopterus_annectens	1	0	7	673	681
neoFor_v3.1	Neoceratodus_forsteri	1	0	5	531	537
aRhiBiv1.1	Rhinatrema_bivittatum	1	0	2	355	358
UCB_Xtro_10.0	Xenopus_tropicalis	1	1	0	689	691
AmbMex60DD	Ambystoma_mexicanum	1	0	0	226	227
"This study"	Pleurodeles_waltl	1	0	0	320	321
ASM2665232v1	Pleurodeles_waltl	1	0	0	307	308

Table S7. The gene numbers of taste receptor type 1 genes (T1R).

Assembly ID	Species name	T1R1	T1R2A	T1R2B	T1R3A	T1R3B	T1R4	T1R5	T1R7	T1R8	Total
ASM1683550v1	Polypterus_senegalus	1	1	1	0	1	1	0	0	1	6
LatCha1	Latimeria_chalumnae	0	2	0	0	2	1	3	0	0	8
PAN1.0	Protopterus_annectens	0	0	0	1	0	3	4	1	0	9
neoFor_v3.1	Neoceratodus_forsteri	1	0	0	1	0	4	3	0	0	9
aRhiBiv1.1	Rhinatrema_bivittatum	1	1	0	0	2	1	1	0	0	6
UCB_Xtro_10.0	Xenopus_tropicalis	0	0	0	0	0	0	0	0	0	0
AmbMex60DD	Ambystoma_mexicanum	1	1	0	2	2	2	1	1	0	10
"This study"	Pleurodeles_waltl	1	1	0	2	2	1	1	1	0	9
ASM2665232v1	Pleurodeles_waltl	1	1	0	2	2	1	1	1	0	9

Supplementary Figures

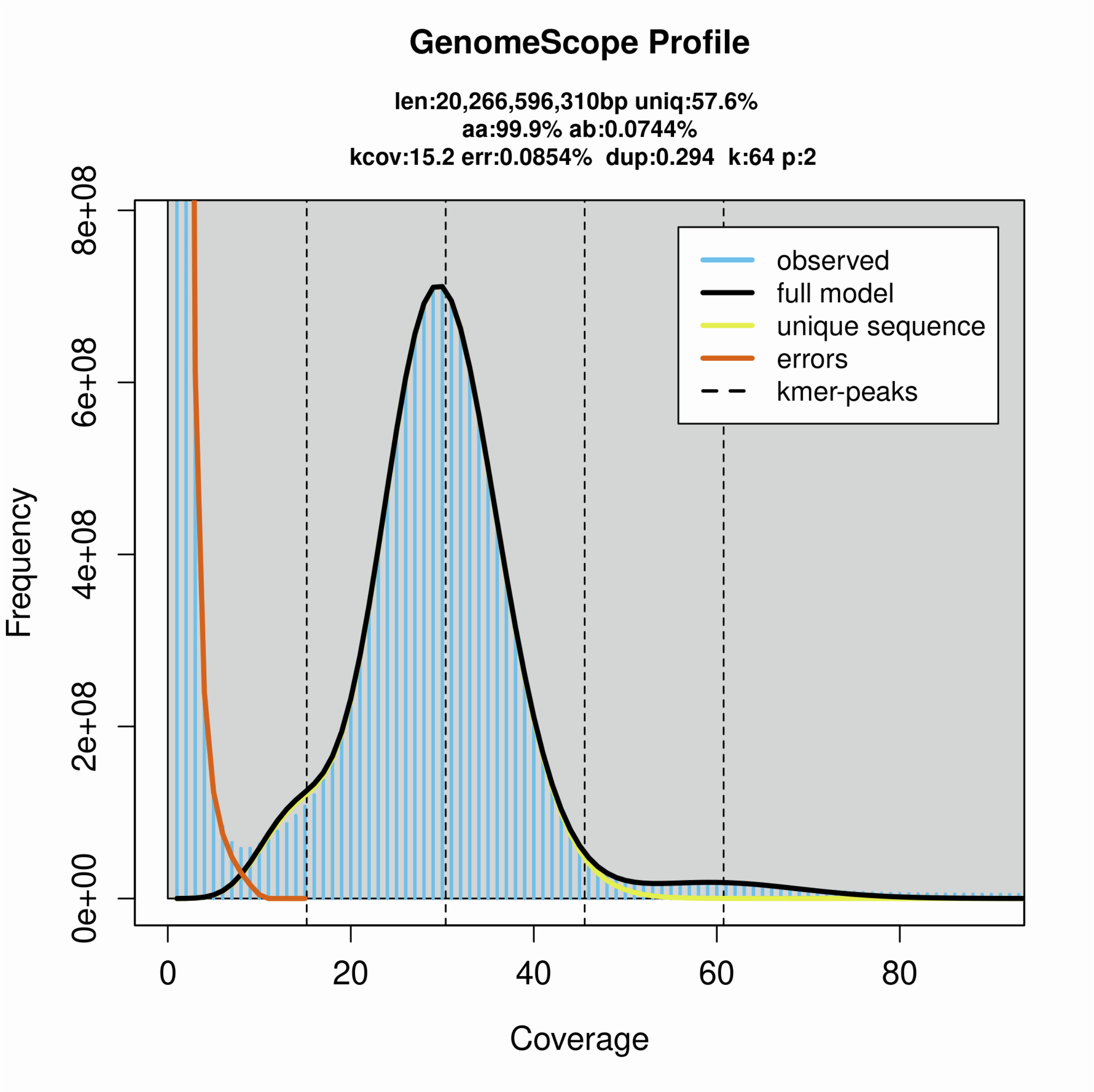

Figure S1. **k-mer plot of HiFi-reads.** k-mer analysis was conducted using Merqury v1.3 and GenomeScope2 pipeline. Because of the large genome size, k-mer = 64 was used for analysis.

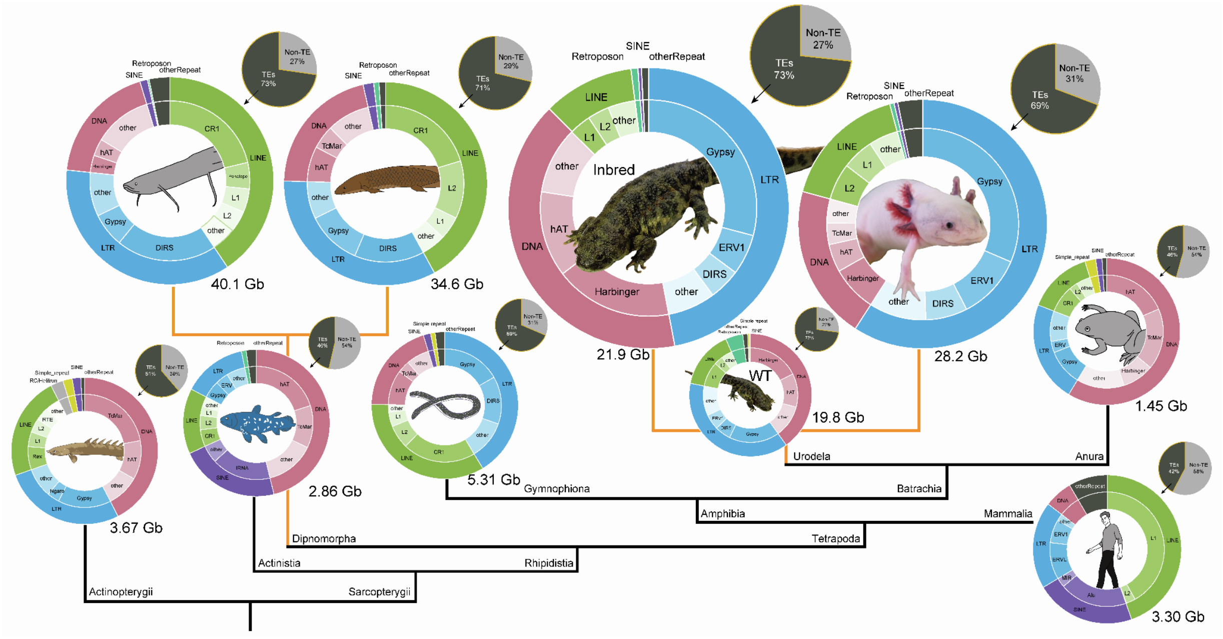

Figure S2. Genomic TE composition in the genomes and phylogenetic relationships among species. For each species, the upper right pie chart shows proportions of transposable elements (TEs) in the genomes. The center doughnut chart shows proportions of major TEs (DNA, class II transposon; LTR, long terminal repeats; LINE, long interspersed nuclear elements; Retroposon, class I transposons, which were classified neither LTR, LINE and SINE; SINE, short interspersed nuclear elements; other repeats (e.g., simple repeat, satellite, Helitron). The inner doughnut chart shows major proportions of superfamilies. Genome sizes based on FASTA data are shown in the lower right corner of the chart. Species with enlarged genome sizes are indicated by orange branches. Species in the figure are, from left to right, *Polypterus senegalus*, *Protopterus annectens*, *Latimeria chalumnae*, *Neoceratodus forsteri*, *Rhinatrema bivittatum*, *Pleurodeles waltl* (our incipient inbred line and the wild type), *Ambystoma mexicanum*, *Xenopus tropicalis*, and *Homo sapiens*.

The major elements of repetitive elements in the inbred line newt genome were LTR/Gypsy (16%), DNA/Harbinger (13%), and DNA/hAT (9%). We compared these results with the axolotl genome, applying the same analysis, and obtained a repetitive sequence content of 69.2%, comprising LTR/Gypsy (22%), DNA/Harbinger (5.4%), and DNA/hAT (3.3%). The repetitive sequences in the axolotl and *P. waltl* genomes were both occupied by LTRs, which consisted of Gypsy, ERV1 and DIRS. While Harbinger and hAT are predominant in the DNA transposon family, their proportion in the genomes varied between the two species, with 13% and 9% of the genome composed of Harbinger and hAT respectively in *P. waltl*, and 5.4% and 3.3% respectively in the axolotl.

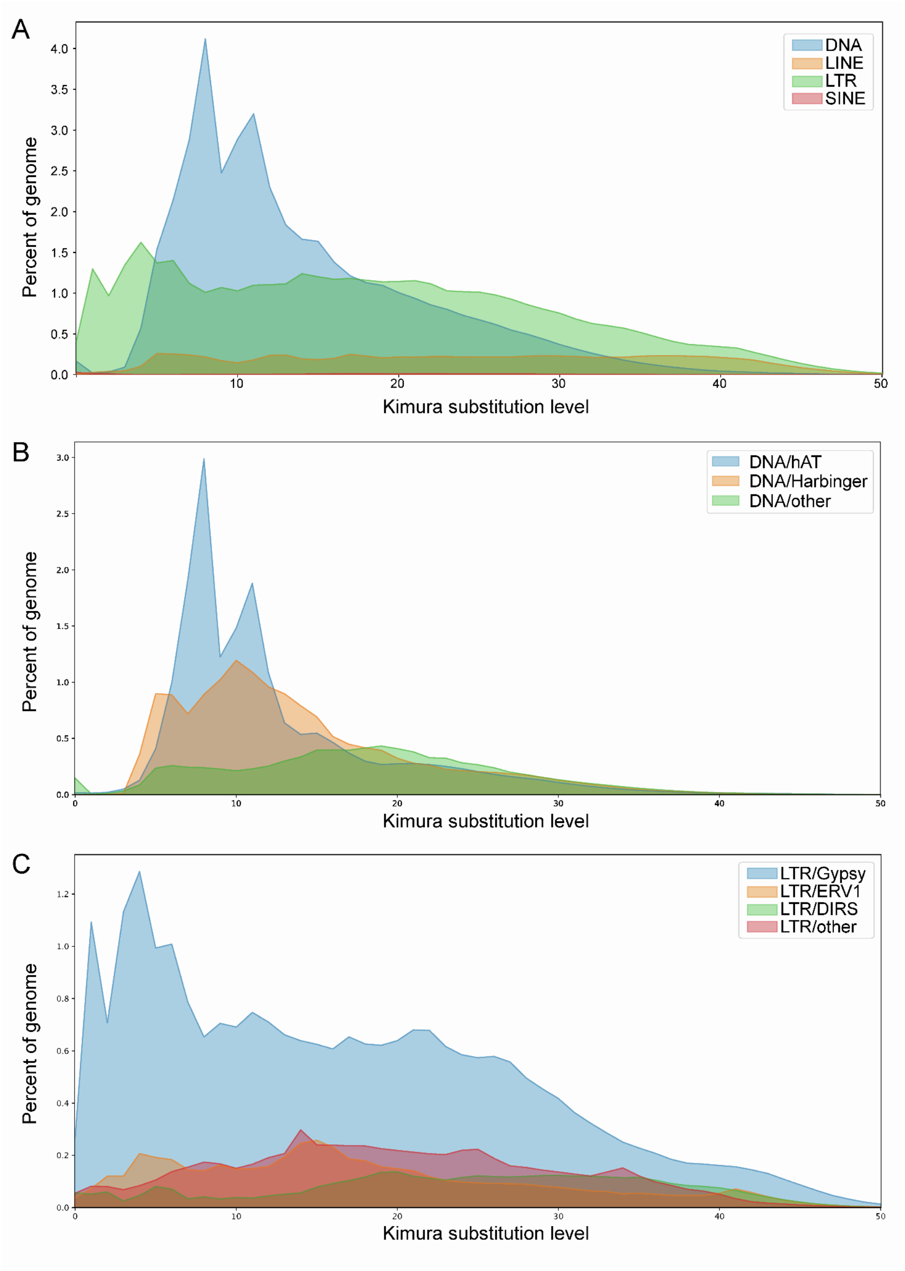

Figure S3. **The landscapes of transposable elements of our incipient inbred *P. waltl* line.** (A) The area chart shows repeat landscapes of the main order of TEs: DNA transposon (blue), LINE (orange), LTR (green) and SINE (red). (B) The area chart shows DNA transposons landscapes: hAT (blue), Harbinger (orange) and the others (green). (C) The area chart shows LTR transposons landscapes: Gypsy (blue), ERV1 (orange), DIRS (green) and others (red). The landscapes of LTR, LINE, and SINE, which were elucidated by the divergence from calculated consensus sequences based on the Kimura substitution model, suggested that they were amplified continuously. In contrast, the landscape of DNA transposons showed an evident peak (A), which was represented by hAT TEs (B). The primary component of the LTR transposon peak was explained by the expansion of Gypsy (C).

A

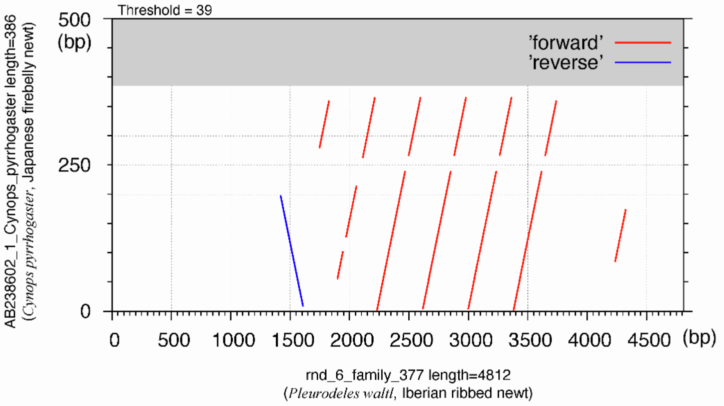

B

```
Pwa-ST-Bam   -ttttttgcaaagtgaacacctgtggattttggcctcttgcacagccggcaactacagaa
CYP-ST-BamB2 ---ttttctgcagtgcctagctgtagattttgaaccctagctctgcgggcacctagggaa
CYP-ST-BamB1 ttttttggcaaagtgcctagctgtagtttttttaaccctagctctaccggcacaaagggaa
CYP-ST-BamA1 -tgttttgcaatatgacaaaatgtggatgttgaaccctagctcagtcggacctaggtta
CYP-ST-BamA2 -ttttttgcaatatgcctagctgtggatgttgaacacttgctcagtcggacctaggtta
TVm8         -gggggtggggggggtcacctg-ggttttggccccgtgctcagctgccagctagggaa
                           *   *  **  * * **   * *  ** *    *  *   *    *
Pwa-ST-Bam   acctaccaaaccagtgcattttgaaaactagagaccgagtggaatccaagatggggtga
CYP-ST-BamB2 acctagcaatcctatacattttgaaaactagacacccaggcaaatcaaggatggggtga
CYP-ST-BamB1 acctagcaaacctatacattttaaaaactagacacccaggggaatccaggatggggtga
CYP-ST-BamA1 acctagaaaacctatacattttgaaaactagacatccaggggaacctaggatgtgggga
CYP-ST-BamA2 acctagcaaacctatacattttttaaaacaagacatccaggggaatccaagaaggagggt
TVm8         acctaccaagcccacacatttctgaaaactagacacccgggggatttcagggaggtatgg
             *****  ** **    ***** * ***** *** * *  *   *    * *  *    *
Pwa-ST-Bam   cttgtggggctctgaccaggttctgttacccaaaatcctttgcaaacctcaaaatgtggc
CYP-ST-BamB2 cttgtctggctctcaccaggttctgttacccagaatcctttgcaaacctcaaactttgta
CYP-ST-BamB1 cttgactgcatcccaccaggttctgttacccagaatcccttgcaaactctaaaaaattgt
CYP-ST-BamA1 cttgtgtggttttcaccaggttctgttacccagaattcctatcaaaccgcaaagtttgt-
CYP-ST-BamA2 cgtgtgtggttttcacaaggtgctgttacccagaatcccttgcaaacctcaactttg--
TVm8         cttgtgtggatcccccaacatttcgtacccagaatcccttgcaaacctcaaatttagc-
             * **   *  *    * *  *  *  ****** *** * *  *****   **
Pwa-ST-Bam   caaaaaaa-cacttttcgacacatttc-gtgacagaaagttctggactctaag------
CYP-ST-BamB2 aaaaaaaaccacattttcctcacatttcaatggtggaaagttctggaatctgcgtggatc
CYP-ST-BamB1 caaaaaaaccacattttcaccacatttctgtggtggaaagttctggaatctgtgtggatc
CYP-ST-BamA1 aaaaaaaaacacattttcctaacatttctgtgatagaaacttctggaatctacgtggatc
CYP-ST-BamA2 aaaaaaaaacacattttcctcactttctgtgatagaatcttctggaatctgggtggatc
TVm8         taaaaaaa--tcattttccacacattttgtgtgggaaagttgtggcatcaccg------
              *******   * *****   ** ***   **   ***  ** ***  **   *
Pwa-ST-Bam   aggagccacaaatttccttacacccagcgttccctcaagtctcccgataaaaataatacc
CYP-ST-BamB2 cggatccaccaaattccacccaccc-gcattccccaaagtcttccgagaaaaatggtacc
CYP-ST-BamB1 cggatccacaaatttccactcaccccgtgttccccaaagtcttccgagaaaaatggtacc
CYP-ST-BamA1 cggatcctcaaactttcacacaccctgcattccccatagtctcctgataaaggtggtatc
CYP-ST-BamA2 cggatcctcaaactttcacacaccctgcattccccatagtctcccgataaagaatgtatc
TVm8         ggctgacactaatttcctaccacccagcgttcctccaagtctctcaataaaattggtgca
              *    * * ** ** *   ***** *  ****    *****    * ***     *
Pwa-ST-Bam   tcacttgtgtgggtagggcttgtgcccgcgacaggaaaagccccaaaacacaacgtgaac
CYP-ST-BamB2 tcacttgtgtgggtaggcctagtgcccacgacaggaaacggcccaaaacacaacgttgac
CYP-ST-BamB1 tcacttgtgtgggtcggcctagtgcctgtgacaggaaacggcacaaaacgcaacgtggtc
CYP-ST-BamA1 tcacttgtgtgggtaggccttgtgcctgccacaggaaatggcacggaacacaacgtggac
CYP-ST-BamA2 caacttgtgtgggtagaccttctgcccgccacaggaaatgtcacggaacacaacgtggac
TVm8         tcactcatgtaggaggcccaagtacccgcaacaggcaagggcccaaaacgtaataagtcg
               ***  *** **  *  *   * **    ***** ** * * *  ***  **
Pwa-ST-Bam   acatcacattttacacagaaaacagaggg
CYP-ST-BamB2 acaccacattttcatagtgaaaactgacct
CYP-ST-BamB1 acacctcaatttcgcagtgaaaaatgaccc
CYP-ST-BamA1 acatcatgattttgcaatgaaaactgaccc
CYP-ST-BamA2 acatcatattttgcaatgaaaactgaccc
TVm8         acaataaaattggcccatcacaaaacaacc
             ***      **        * **   *
```

Figure S4. **A newt-specific microsatellite DNA.** (A) The BLAST hits between the two newt sequences. The 'forward' sequences are plotted in red and the 'reverse' sequences in blue with reference to the microsatellite DNA of the Japanese fire-bellied newt *Cynops pyrrhogaster* (Cyp-ST-Bam). The horizontal axis shows the library sequence with *P. waltl*. Approximately 5 microsatellites are contained within the rnd_6_family_377 library sequence of *P. waltl* (Pwa-ST-Bam). (B) Comparison of newt-specific satellite DNAs among three species, *P. waltl* (Pwa-ST-Bam), *C. pyrrhogaster* (CYP-ST-Bam) and *Lissotriton vulgaris* (TVm8).

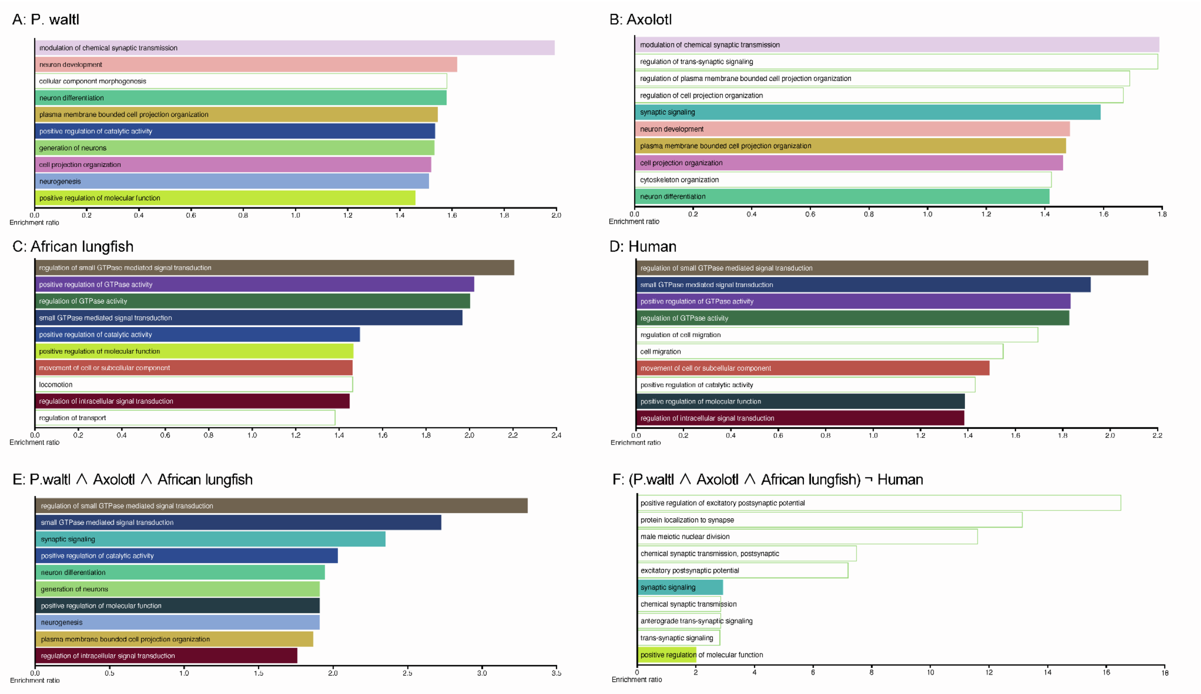

Figure S5. **Results of enrichment analysis of genes containing the top 5% long introns.** Enrichment analysis of genes with introns in the top 5% of intron lengths in *P. waltl* (A), Axolotl (B), African lungfish (C), Human (D). Enrichment analysis of genes common in axolotl, *P. waltl*, and African lungfish (E) and (E) minus genes with introns in the top 5% of human length (F). In all plots, the same color is assigned to the same GOs. White bars have nothing in common with any of the other bars. All FDR ≤ 0.05

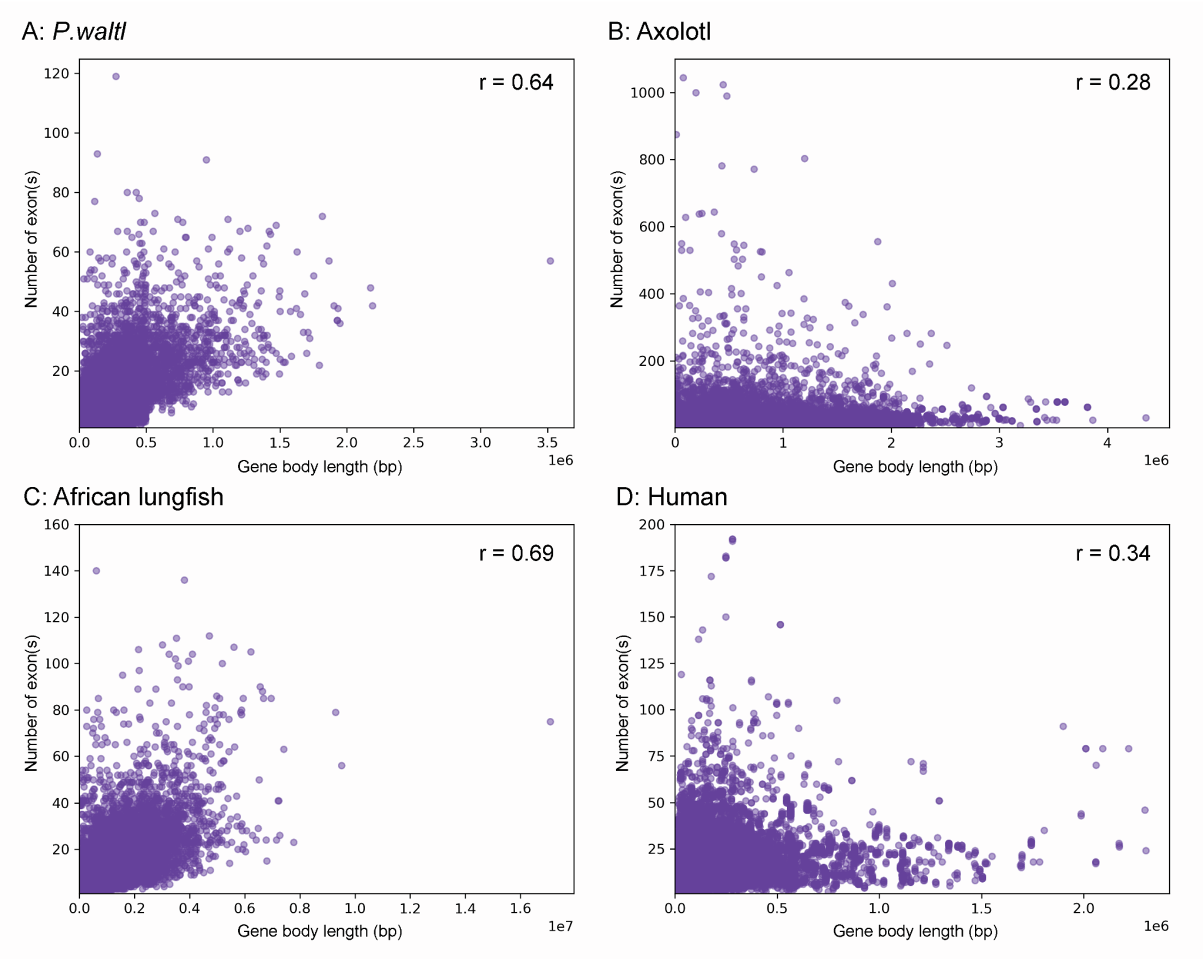

Figure S6. **Correlation between gene length and exon number.** Plots show gene length (bp) vs. the number of exons in the gene. (A) *P. waltl*, (B) Axolotl, (C) African lungfish, and (D) Human. Correlation coefficients are shown in the upper right corner.

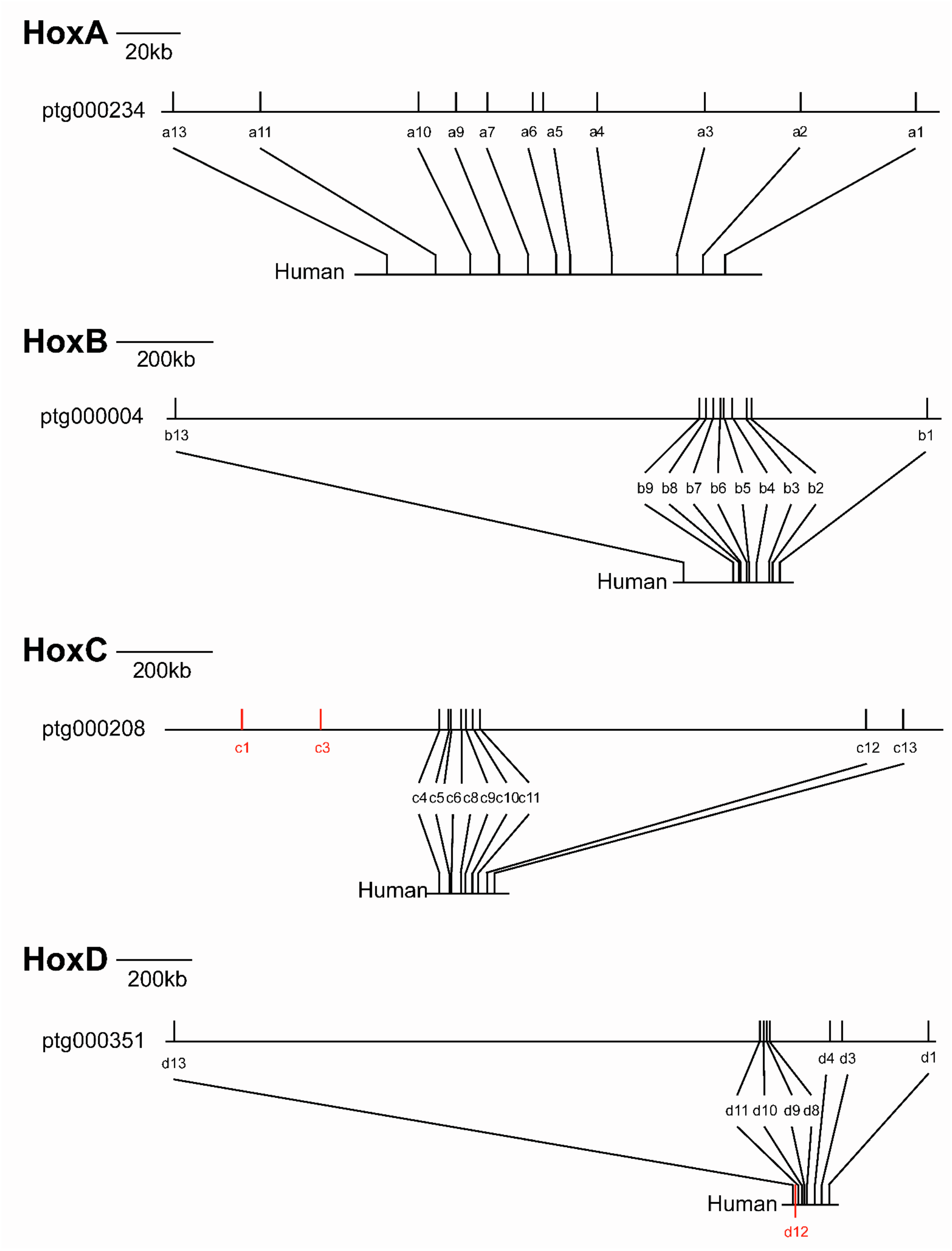

Figure S7. Comparison of Hox cluster sizes between *P. waltl* and human. *P. waltl*'s genome harbors four Hox clusters (*HoxA*, *HoxB*, *HoxC* and *HoxD*), containing a total of 40 genes. It is known that *Hoxc1* has been largely lost in the tetrapod lineage, *Hoxc3* is not present in mammals and *Hoxd12* is not present in amphibians. Remarkable expansions were observed in the Hox clusters, particularly in HoxB, HoxC, and HoxD, in comparison to the human genome. The longest expansion lengths within each cluster are observed as follows: 46.0 kb between *Hoxa10* and *Hoxa11*, 1085.0 kb between *Hoxb9* and *Hoxb13*, 800.1 kb between *Hoxc11* and *Hoxc12*, and 1556.0 kb between *Hoxd11* and *Hoxd13*.

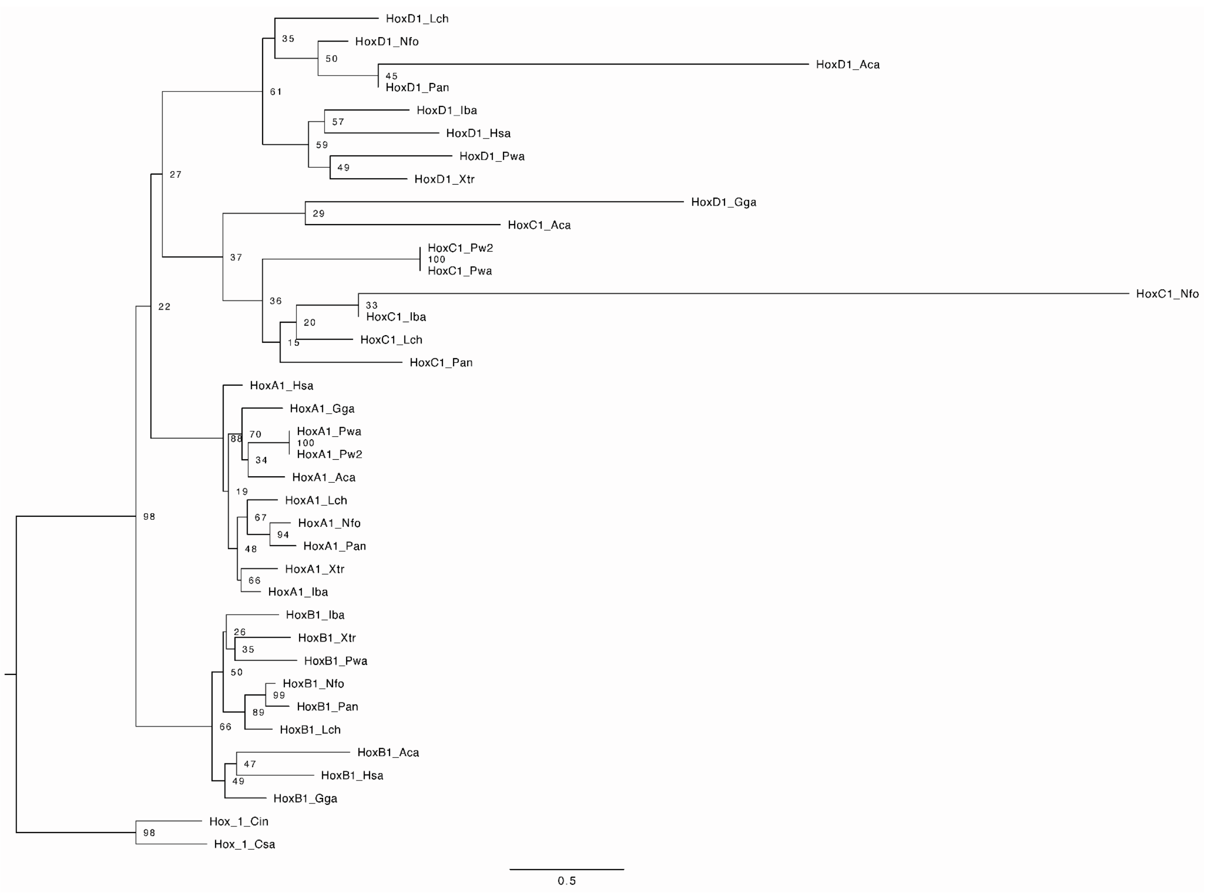

Figure S8. Molecular phylogenetic tree of Hox1 genes. The *Hoxc1* gene of *P. waltl* is retained in the genome. Amino acid sequences of HoxC1_Pwa and HoxC1_Pwa2 were obtained by translating the cDNAs based on the genome and transcriptome annotation, respectively.

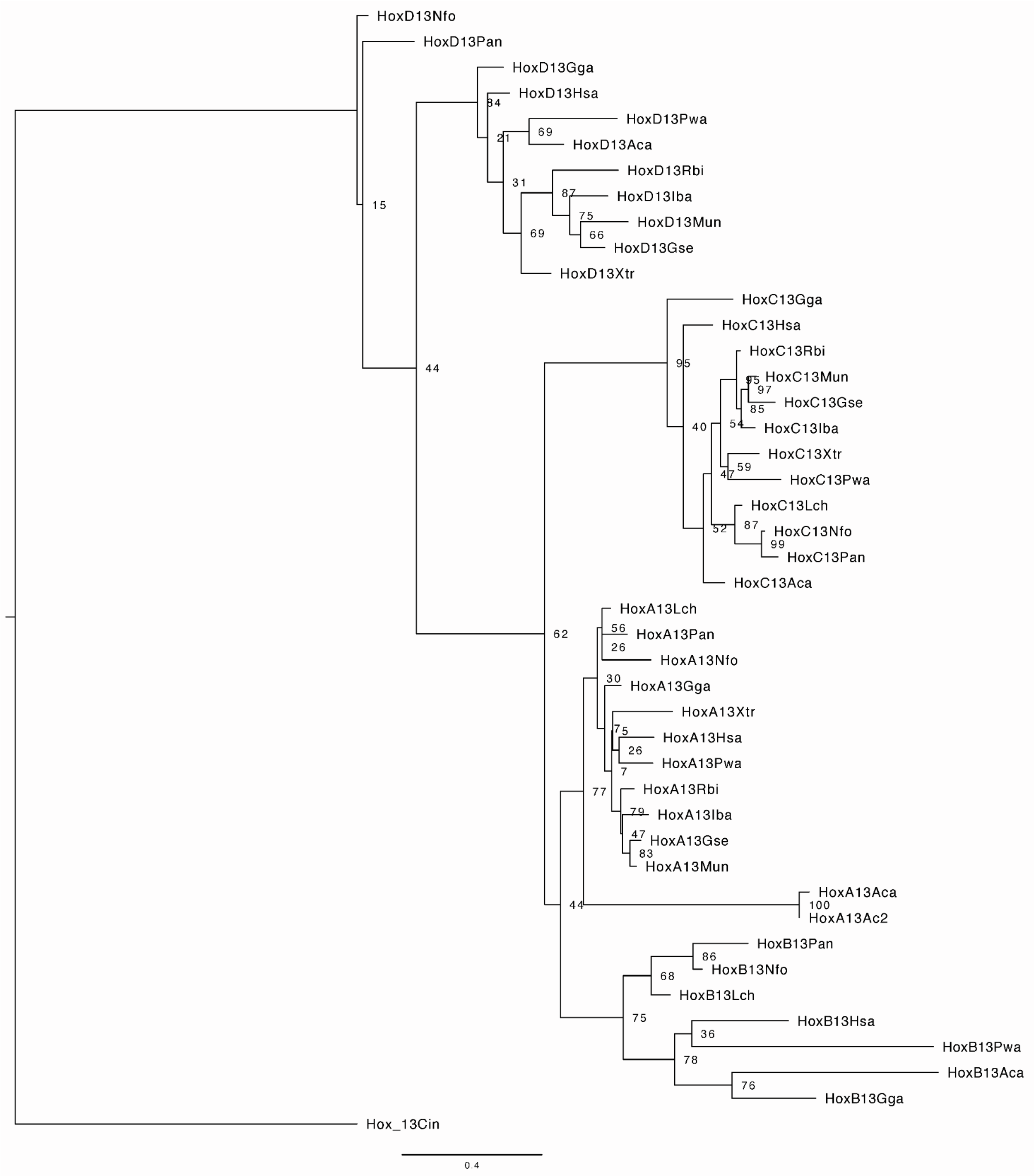

Figure S9. Molecular phylogenetic tree of Hox13 genes. The *Hoxb13* gene of *P. waltl* is retained in the genome.

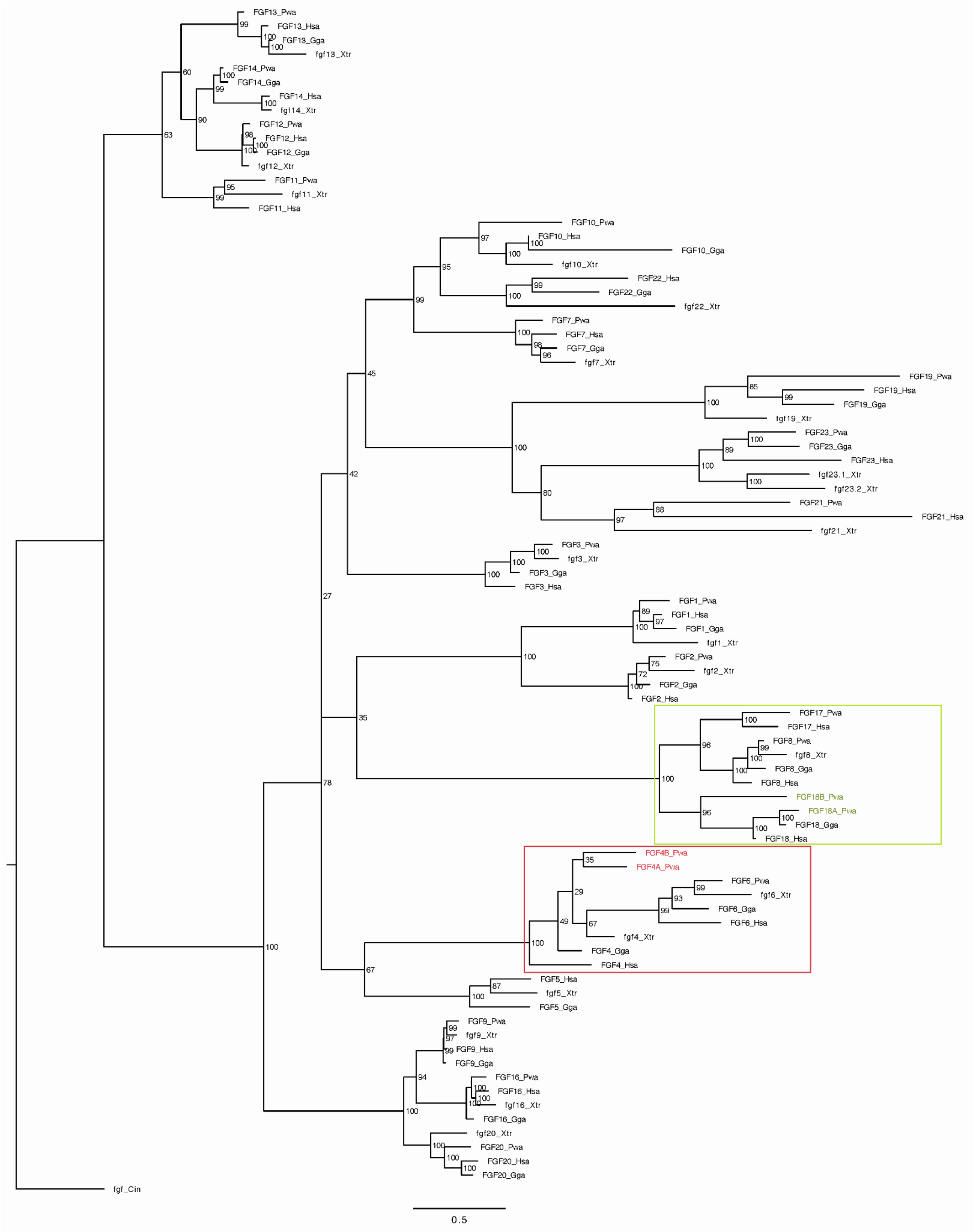

Figure S10. A phylogenetic tree of the Fgf gene family. Consistent with a previous report,[12] we were unable to identify the *Fgf5* gene in our genome data.

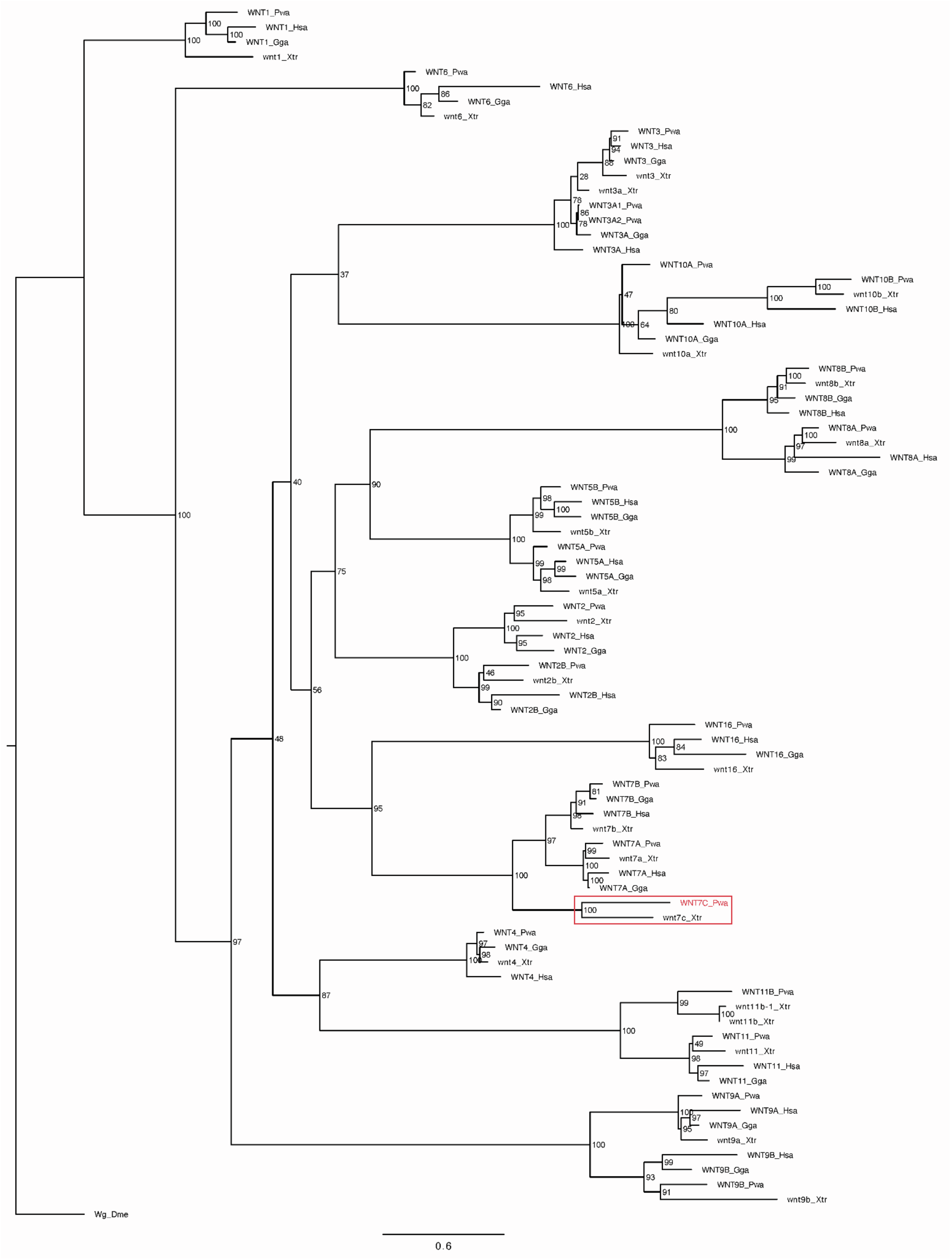

Figure S11. A phylogenetic tree of the Wnt superfamily.

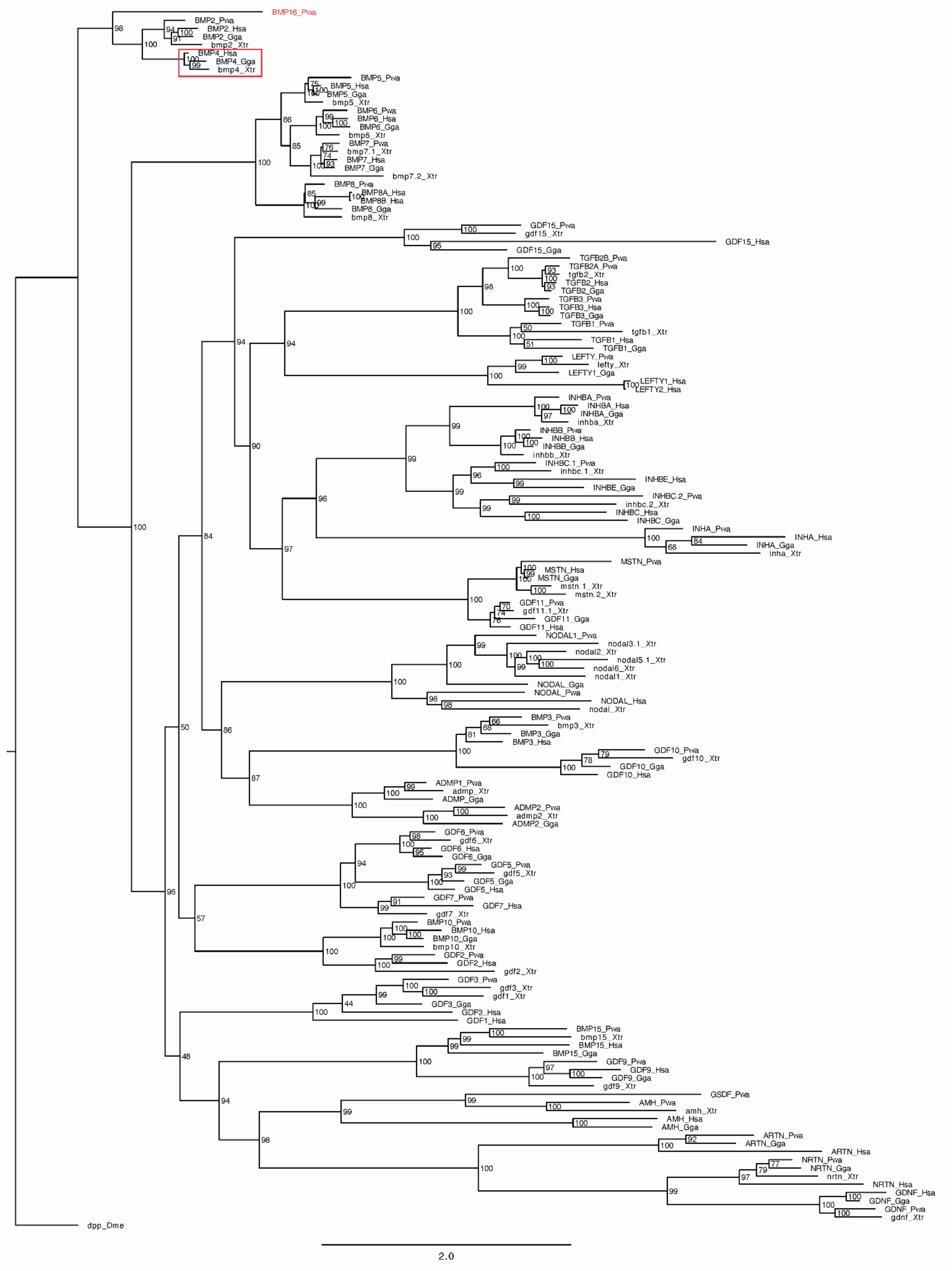

Figure S12. A phylogenetic tree of the Tgf-β superfamily.

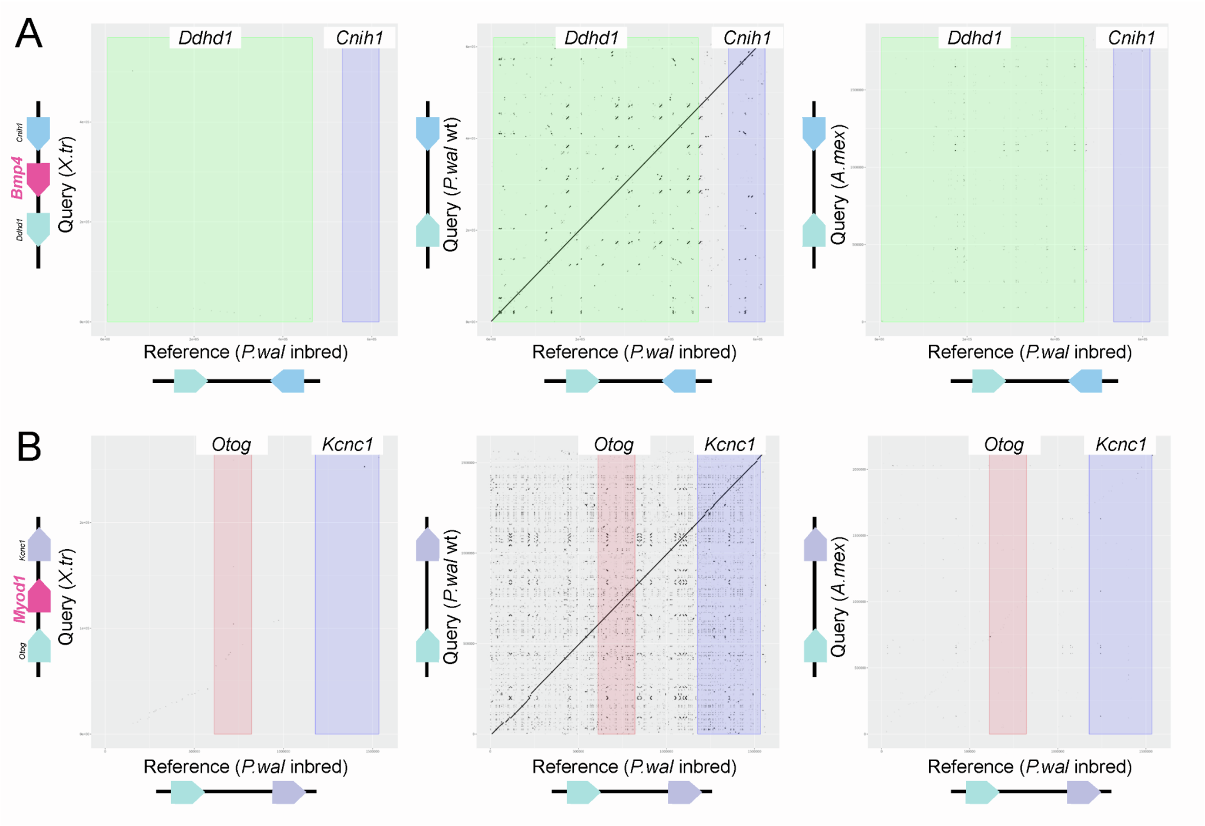

Figure S13. Conserved synteny flanking the *BMP4* and *MYOD1* loci with their absence in *P. waltl* and *A. mexicanum*. Dot plots comparing syntenic regions around *BMP4* (A) and *MYOD1* (B), using the *P. waltl* inbred genome as reference. All-against-all blastn search between reference and each query was conducted. Queries include *X. tropicalis* (*X.tr*), wild-type *P. waltl* (*P.wal* wt), and *A. mexicanum* (*A.mex*). Only hits showing less than 0.1 e-value were plotted. In both loci, the flanking genes (*DDHD1*–*CNIH1* for *BMP4*; *OTOG*–*KCNC1* for *MYOD1*) are conserved across species, whereas *BMP4* and *MYOD1* (magenta) are present only in *X. tropicalis* and absent in *P. waltl* and *A. mexicanum*. Colored regions indicate orthologous genes: green for *DDHD1*, purple for *CNIH1* (A), red for *OTOG*, and purple for *KCNC1* (B). Schematic gene models are shown beside and below each plot.

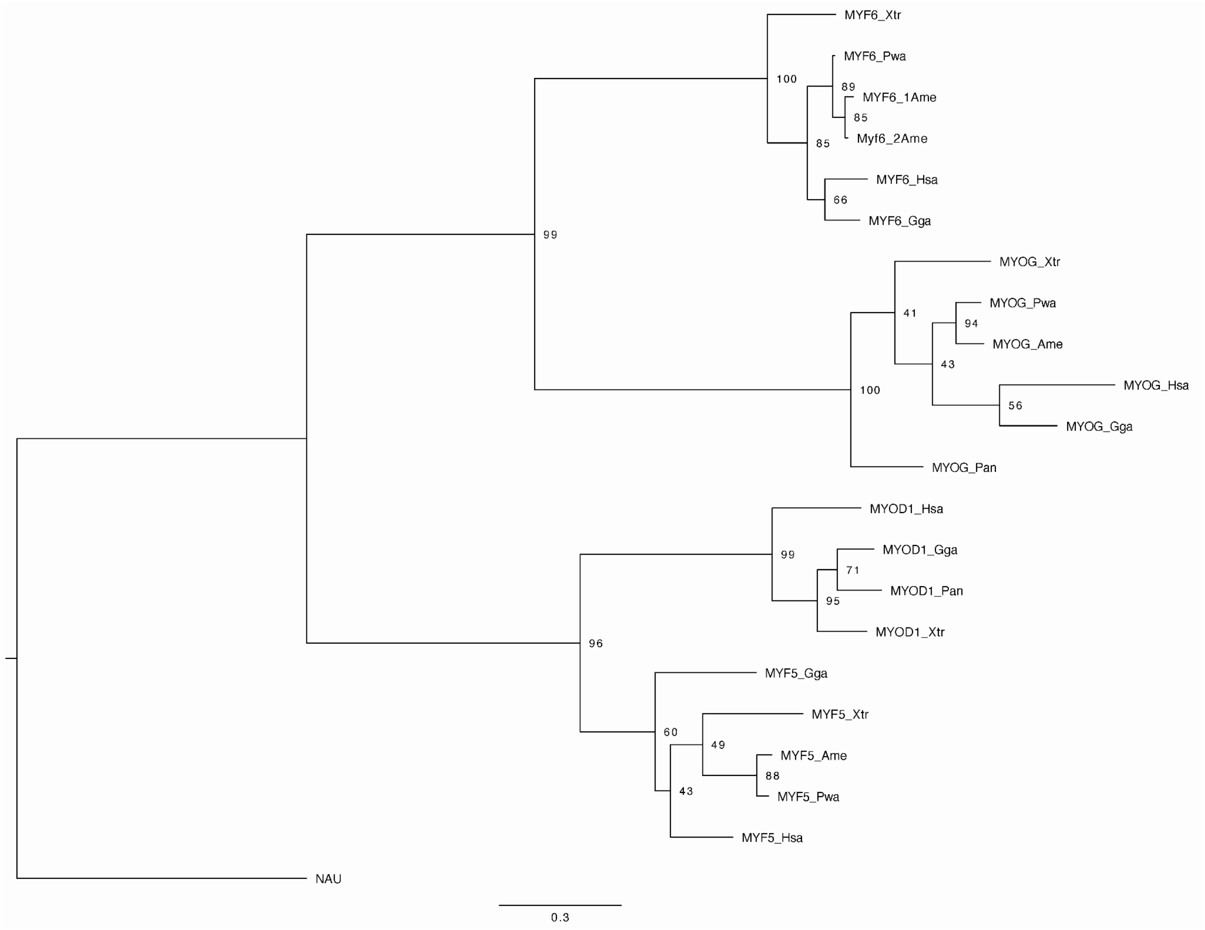

Figure S14. A phylogenetic tree of myogenic regulatory factors (*Myod1*, *Myf5*, *Myf6*, and *Myogenin*) in vertebrates (Hsa, Gga, Xtr, Ame, Pan and Pwa). NAU, a *Myod* homolog in *Drosophila*, is used as an outgroup. Note that *Myod1* genes are absent in salamanders (see Figure 3B). Note that, using the *P. waltl Myf5* sequence as a query, we searched the African lungfish (*Protopterus annectens*) genome with TBLASTX under default parameters but detected no orthologs above the significance threshold.

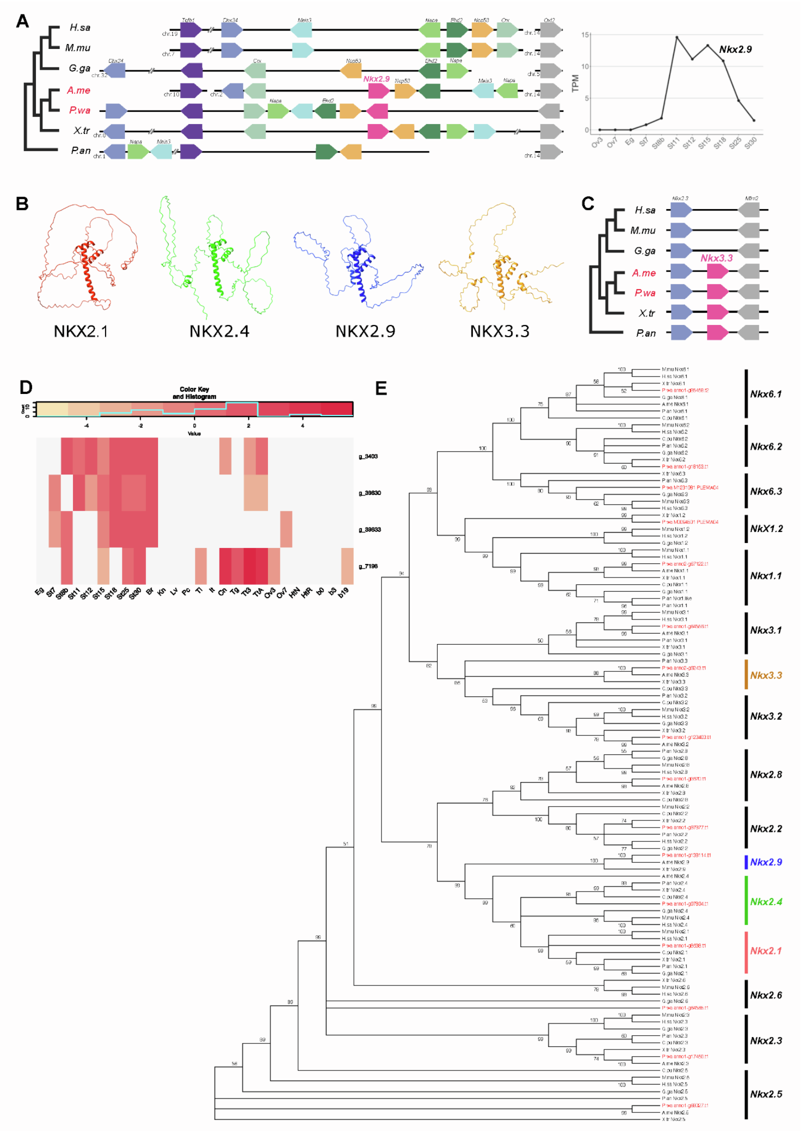

Figure S15. Synteny, expression, protein structure, and phylogeny of *Nkx* genes. (A) Retention of amphibian-specific *Nkx2.9* (left) and its expression in developmental stages (right). (B) The estimated structures of NKXs based on AlphaFold2. (C) The synteny of *Nkx3.3* genes, which are not found in the genomes of humans, mice, and chicken. (D) Log-transformed expression levels of g_3403(*Nkx3.3*), g_39630(*Nkx2.9*), g_39633(*Nkx2.4*), g_7196(*Nkx2.1*) in developmental stages and each tissue; Br: brain, Kn: kidney, Lv: liver, Pc: pancreas, Tl: tail, It: intestine, Cn: connective tissue, Tg: testicular gland, Tt3: testis (3 months juvenile), TtA: testis (adult), Ov3: ovary (3 months juvenile), Ov7: ovary (7 months juvenile), HtN: heart (ventricle, normal), HtR: heart (ventricle, regenerating), b0: limb (normal), b3: limb (3 days post-amputation), b19: limb (19 days post-amputation). Data points with zero counts were colored white. (E) The phylogenetic tree of *Nkx* genes in vertebrates. The *Nkx* genes of *P. waltl* are labeled in red.

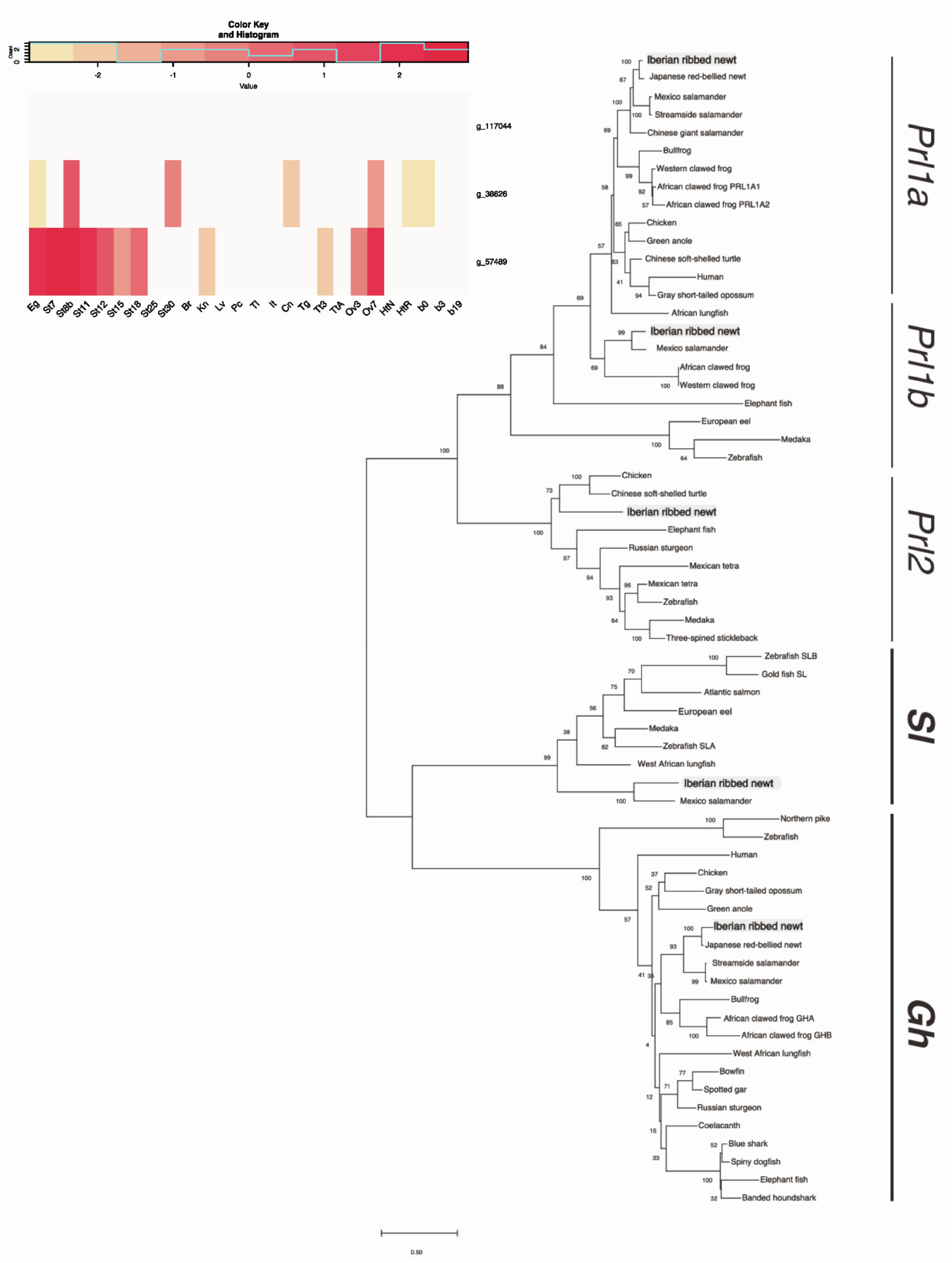

Figure S16. Expression and phylogenetic tree of *Prl1a*, *Prl1b*, *Prl2*, *Sl*, and *Gh*.
Log-transformed expression levels of g_117044(*Prl1a*), g_36626(*Gh*), g_57489(*Sl*) in developmental stages and each tissue; Br: brain, Kn: kidney, Lv: liver, Pc: pancreas, Tl: tail, It: intestine, Cn: connective tissue, Tg: testicular gland, Tt3: testis (3 months juvenile), TtA: testis (adult), Ov3: ovary (3 months juvenile), Ov7: ovary (7 months juvenile), HtN: heart (ventricle, normal), HtR: heart (ventricle, regenerating), b0: limb (normal), b3: limb (3 days post-amputation), b19: limb (19 days post-amputation). Data points with zero counts were colored white. The phylogenetic tree of GH/PRL family genes in vertebrates. The *Gh/Prl* family genes of *P. waltl* are labeled in bold and shaded background.

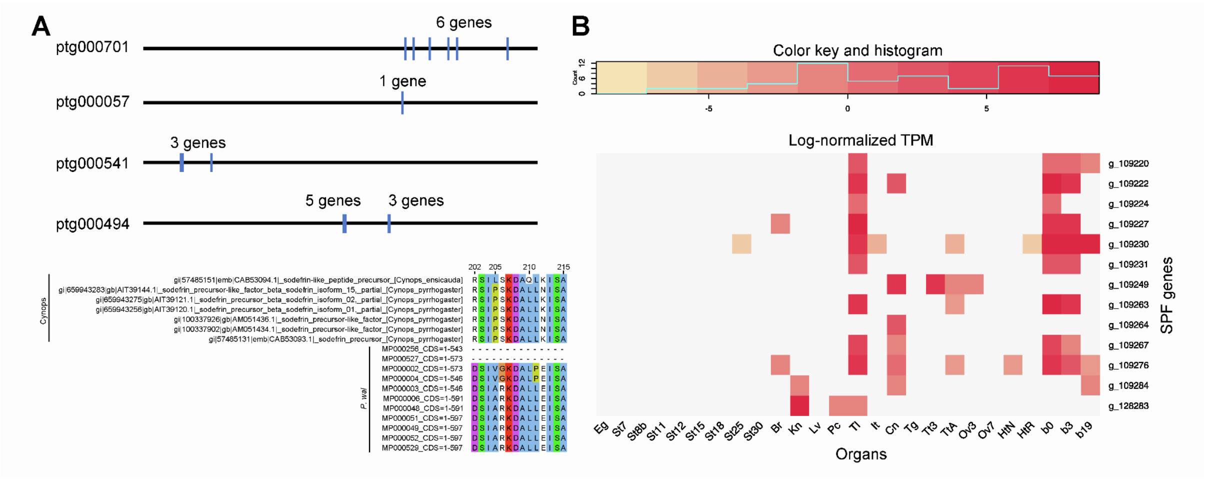

Figure S17. Expression of *Spf* genes in the *P. waltl* genome. (A) Number of *Spf* genes in contigs (top) and alignment with Sodefrin amino acid sequence in *Cynops* (bottom). (B) Log-transformed expression levels of *Spf* genes in each tissue; Br: brain, Kn: kidney, Lv: liver, Pc: pancreas, Tl: tail, It: intestine, Cn: connective tissue, Tg: testicular gland, Tt3: testis (3 months juvenile), TtA: testis (adult), Ov3: ovary (3 months juvenile), Ov7: ovary (7 months juvenile), HtN: heart (ventricle, normal), HtR: heart (ventricle, regenerating), b0: limb (normal), b3: limb (3 days post-amputation), b19: limb (19 days post-amputation). Data points with zero counts were colored white.

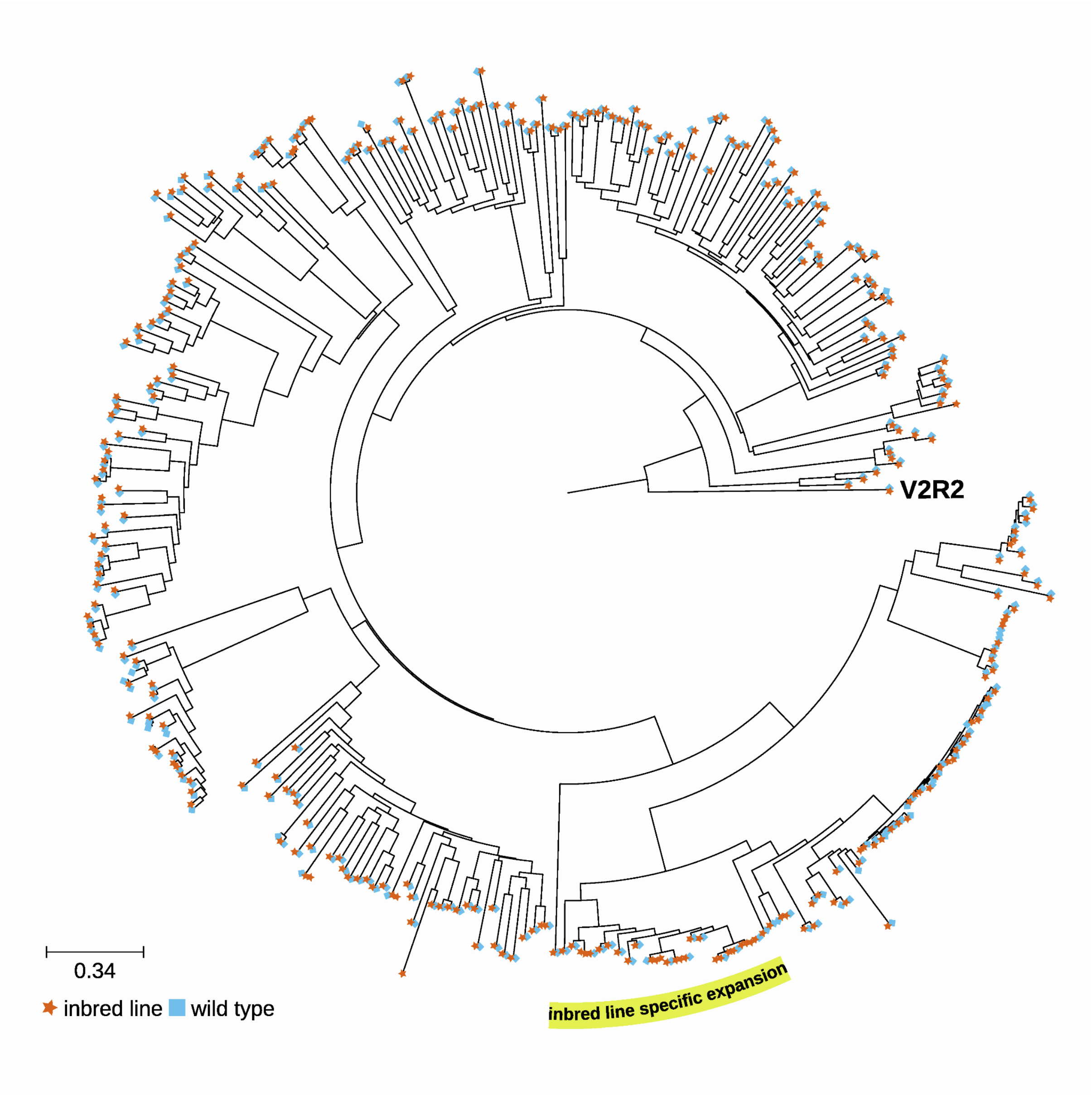

Figure S18. Phylogenetic tree of t-*V2R* genes from both the wild type and our inbred line. The canonical *V2R2* gene was included as an outgroup. t-*V2R* genes from the inbred line genome assembly are indicated by orange stars, and those from the wild-type genome assembly are indicated by blue squares. The scale bar represents the number of amino acid substitutions per site. Note that the *V2R*s duplicated specifically in the inbred line form a monophyletic group.

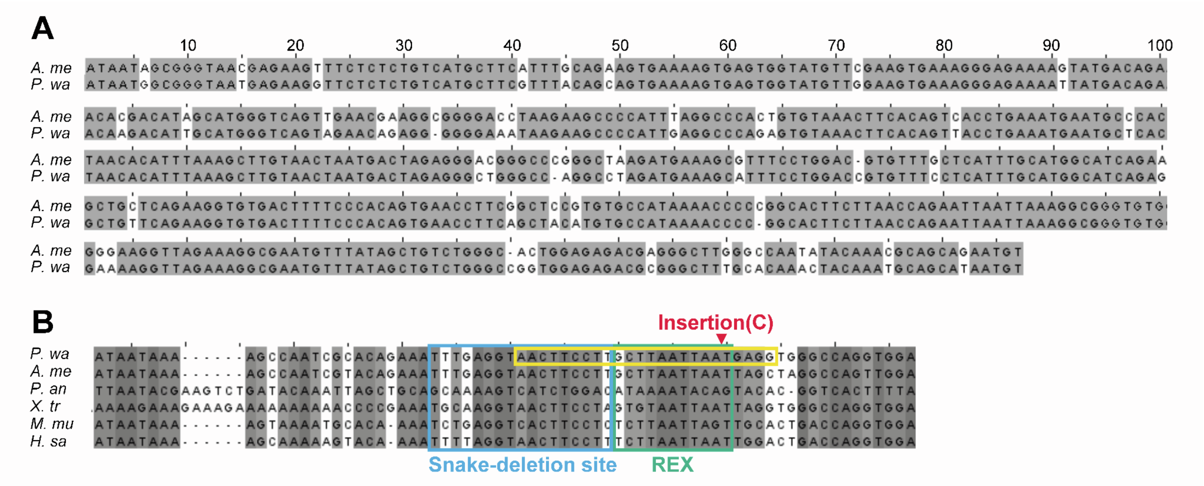

Figure S19. Limb-specific enhancers in *Fgf8* (CNE80) and *Shh* (MFCS1/ZRS). (A) Alignment of the genomic region surrounding *Fgf8*_CNE80 between *A. mexicanum* and *P. waltl*. (B) Alignment of the Snake deletion site and REX sequences in *Shh*_MFCS1/ZRS across vertebrates. The yellow box indicates sgRNA site targeting the snake deletion site involved in the newt MFCS1/ZRS mutant line.[10] The red downward arrowhead indicates the 1-bp insertion site (cytosine) described in Figure 5, and the blue and green boxes denote the snake deletion site and REX sequence, respectively.[60]

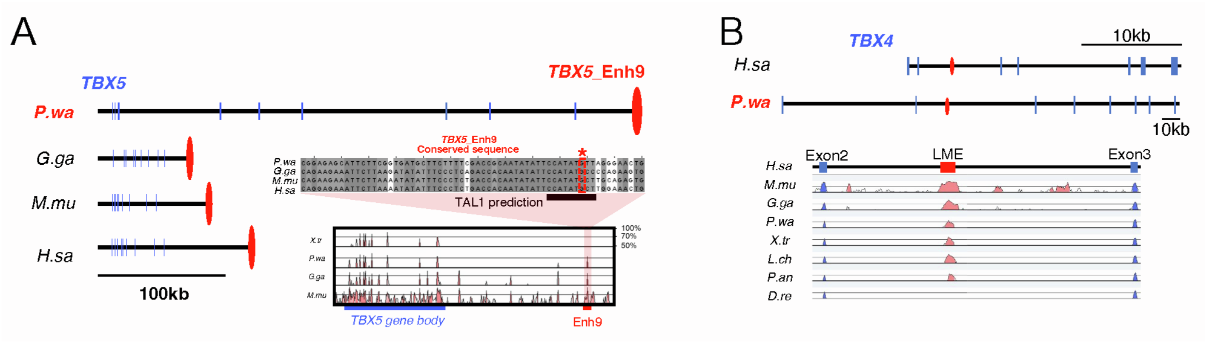

Figure S20. Heart- and lung-specific CNEs. (A) *Tbx5*_Enh9 is implicated in human heart disease. These CNEs exhibit high conservation across vertebrates. (B) *Tbx4*_LME is characterized as a lung mesenchyme-specific CNE. This lung enhancer is located on the intron 3. Note that LME could not be found in the *D. rerio* TBX4 gene body.

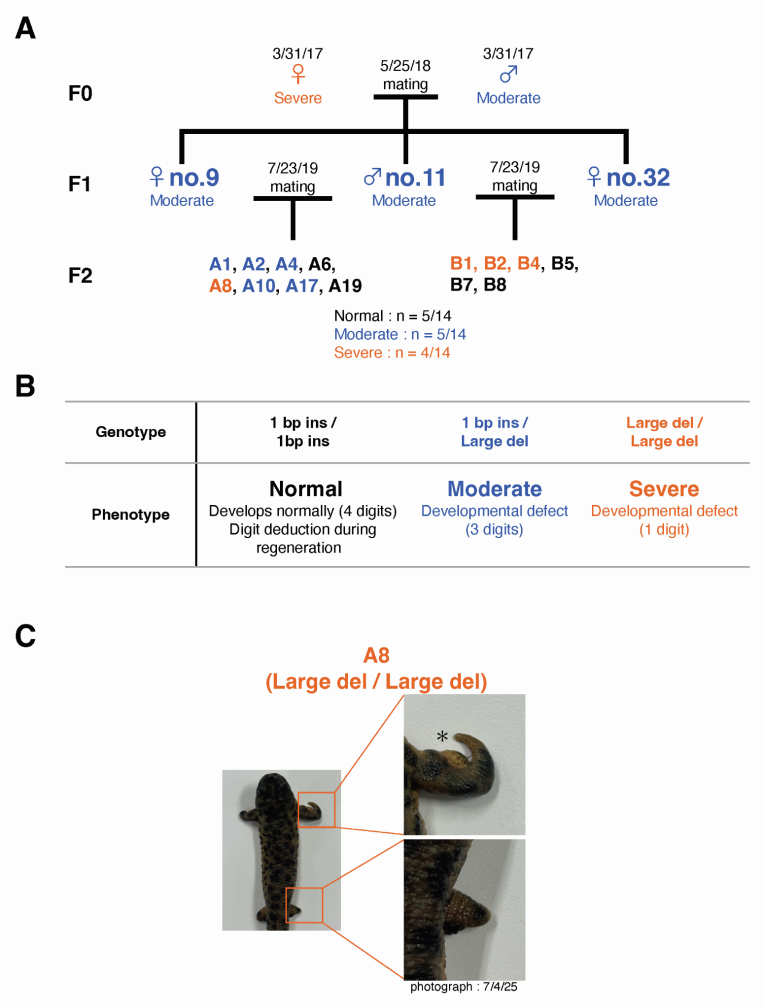

Figure S21. Generation of MFCS1/ZRS mutant lines and their phenotypes in limb development. (A) A schema of pedigree of MFCS1/ZRS mutant newt lines. A gRNA targeting the region flanking the snake deletion on MFCS1/ZRS was used with CRISPR–Cas9 to generate F_0 founders (described in Figure S19 and Suzuki *et al.*, 2018).[10] The F_0 individual exhibiting both severe (1 digit in both fore- and hindlimb) and moderate (three digits in forelimb and four digits hindlimb) phenotypes in limb development was crossed to produce F_1 offspring, which were sibling-mated to yield F_2 progeny. F_2 genotypes segregated into 1 bp ins/1 bp ins and large del/large del homozygotes, and 1 bp ins/large del heterozygotes (large del: 700~800 bp deletion around the snake deletion site). A and B series denote genotyped F_2 newts; 1 bp ins/1 bp ins homozygotes (A6, A19, B5, B7, B8) were used for the regeneration experiments in this manuscript. (B) Genotypes and corresponding limb phenotypes of F_2 newts. The 1 bp ins/1 bp ins homozygotes used in the regeneration experiments (Figure 5) displayed normal fore- and hind limb development without digit loss. (C) Phenotype of large del/large del homozygous F_2 newts approximately six years old (The photograph was taken on July 4, 2025). Similar to MFCS1/ZRS knockout mice, these mutants exhibited severe limb phenotypes.

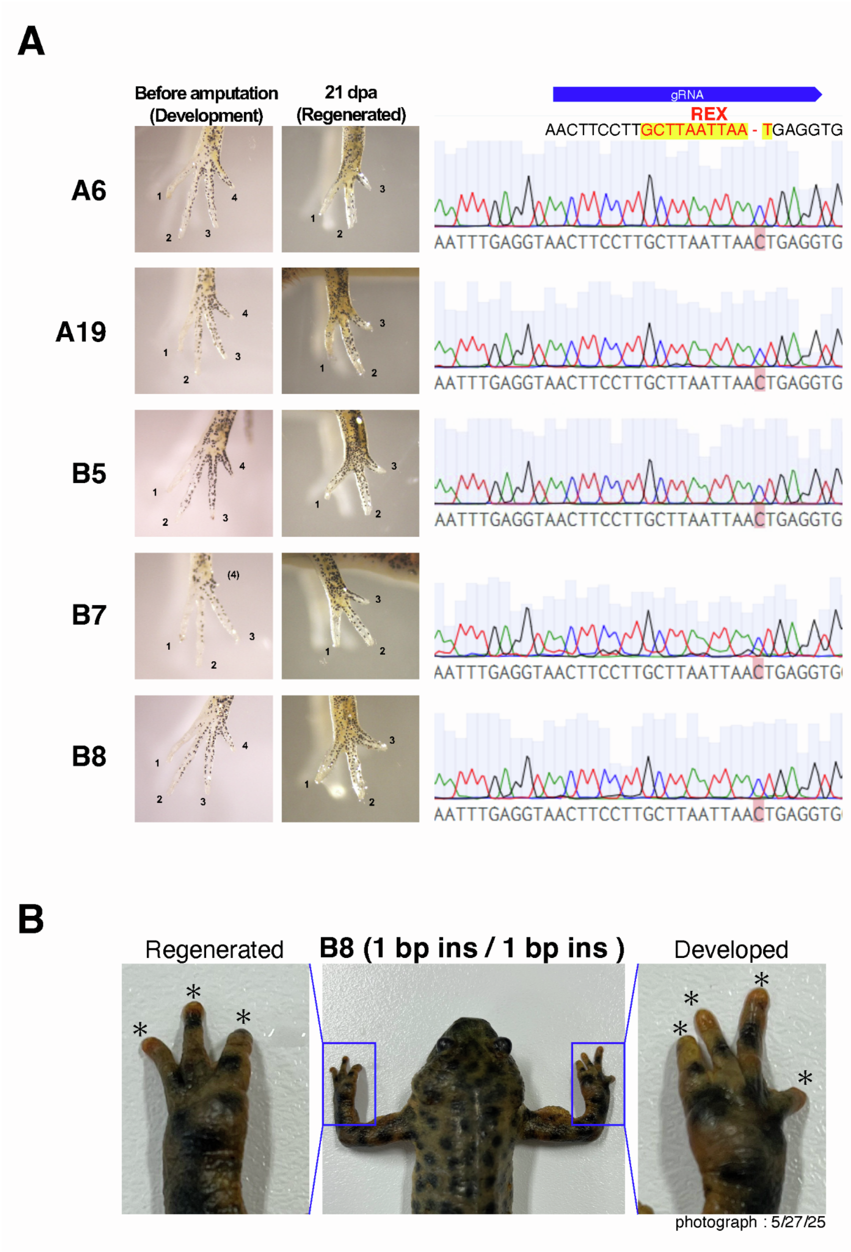

Figure S22. Phenotypes and genotypes of MFCS1/ZRS mutant newts (F_2) with a one-base insertion in regeneration experiment. (A) The left forelimb before amputation and 21 days post-amputation, and sequence of the target site. Homozygous F_2 mutants (1 bp ins/1 bp ins) were obtained from two different clutches (two different F_1 females with the same F_1 male) described in Figure S21. Genotyping results corresponding to each mutant number are shown in the left column. The red-highlighted 'C' indicates the position of a 1 bp insertion in the mutants (see Figure S19). Note that a REX motif is described by a red highlight (Bowser *et al.*, 2025).[60] (B) Photograph of F_2 mutant individual No. B8 approximately six years after the regeneration experiment (The photograph was taken on May 27, 2025). The left regenerated forelimb retained only three digits and never achieved full regeneration to four digits.
